# How Do Firms Respond to Reduced Labor Costs? Evidence from the 2007 Swedish Payroll Tax Reform

**DOI:** 10.1007/s10842-021-00356-6

**Published:** 2021-03-04

**Authors:** Sven-Olov Daunfeldt, Anton Gidehag, Niklas Rudholm

**Affiliations:** 1 Institute of Retail Economics, Stockholm, Sweden; 2 Dalarna University, Falun, Sweden; 3 Örebro University, Örebro, Sweden

**Keywords:** Payroll tax reform,, Labor demand,, Employment,, Wages, H25, H32, J23, J32, L20

## Abstract

One way for policymakers to reduce labor costs and stimulate the recruitment of marginalized groups of labor in a highly unionized economy is to lower payroll taxes. However, the efficiency of this policy instrument has been questioned, and previous evaluations have mostly found small employment effects for such reforms. We investigate the effects of a payroll tax cut in Sweden that decreased firms’ labor costs in relation to the number of young employees that they had employed when the reform was implemented in 2007. We find that most firms received small labor cost savings as a result of the reform, but those that received larger cost savings increased their number of employees significantly more than firms that received no, or minor, labor cost savings. Our findings also suggest that the payroll tax cut increased the total wages paid to incumbent workers, but the wage effect was too small to offset the positive extensive-margin employment effect of the reform. In total, we find that the Swedish payroll tax reform created 18,100 jobs over the period 2006–2008; most of these jobs were within the targeted group of young employees.

**Supplementary Information:**

The online version contains supplementary material available at 10.1007/s10842-021-00356-6.

## **I**ntroduction

Unemployment rates and the number of individuals in neither employment, nor education nor training, are high in many European countries—particularly among first-generation immigrants and young adults with low educational attainment (Papademetriou et al. 2010; Bruno et al. 2014). This development is especially troublesome considering the rising unemployment following the outbreak of the COVID-19 pandemic (Fana et al., 2020). Long periods of unemployment may depreciate individuals’ human capital and can be used as a negative sorting criterion when employers recruit personnel (Lockwood 1991). Long unemployment periods may thus cause persistent high unemployment rates among these groups (Phelps 1972; Heckman & Borjas 1980; Arulampalam 2001), with high economic and social costs as a result.

In highly unionized economies—such as the Scandinavian welfare states—policymakers have limited influence over minimum wages because they are set in negotiations between employer organizations and trade unions. Policymakers tend, under such circumstances, to rely on job subsidies aimed at groups of job seekers having difficulties in entering the labor market (Martin and Grubb 2001). However, these policies have been criticized because they typically are time-limited and can crowd-out regular jobs (Martin and Grubb 2001; Kluve 2006; Nekby 2008). An alternative way for policymakers to reduce firms’ labor costs is to implement payroll tax cuts, but the efficiency of such reforms has been questioned because insiders may use their bargaining power to increase their wages at the expense of outsiders’ possibility to become hired (Holmlund 1983; Gruber 1997).

We investigate the effects of a payroll tax cut of 11.1 percentage points for employees aged 19–25 that was implemented by the Swedish government on July 1, 2007. An important and generally overlooked aspect of the reform was that the payroll tax cut covered all young workers, not only those who were recruited after the reform. Firms that initially had many young employees were thus able to reduce their labor costs substantially due to the payroll tax cut, which means that firms received different doses, or treatment intensities, of the reform based on their number of young employees when the payroll tax cut was implemented. We utilize this firm-level variation in treatment intensity to investigate the effects of the payroll tax reform on the number of employees and total wages of incumbent workers.

Previous studies that have analyzed the labor market effects of the Swedish payroll tax reform in 2007 are inconclusive. Egebark and Kaunitz (2013, 2014) and Skedinger (2014), compared the outcomes for young individuals who were targeted by the Swedish payroll tax cut with the outcomes for slightly older individuals who were not subject to reduced payroll tax. Using this methodology, Egebark and Kaunitz (2013) found that 6–10,000 jobs were created within the targeted age group, suggesting that the payroll tax reform had a fairly small effect on employment and was costly in terms of foregone tax revenues. They found, on the other hand, no indications that the lack of major employment effects could be explained by significant wage spillovers to incumbent workers (Egebark and Kaunitz; 2013, 2017).

However, the fact that the payroll tax cut gave rise to both a substitution effect and a scale effect is ignored when investigating the effects of the reform on individuals just below and above the age threshold. The substitution effect encourages firms to shift towards a more youth labor-intensive production, while the scale effect incentivizes them to expand their production and therefore to increase the usage of other input factors as well. This finding implies that firms might also have spent some of their labor cost savings on recruiting nontargeted individuals, e.g., older and more experienced individuals. The total employment effect can thus differ from the effects previously found for the targeted group of young employees versus slightly older, noneligible employees.

Egebark and Kaunitz (2017) and Saez et al. (2019a) are two recent studies that have acknowledged the link between firms’ labor cost savings and the number of young employees at the time of the reform implementation. However, both studies used relative treatment intensity measures, while we rely on a treatment intensity measured in absolute terms. We believe this distinction to be important because firms’ labor cost savings do not strictly increase with relative treatment intensity measures. For example, if using a relative measure, an employer with three out of four employees below the age of 26 will be defined as being more exposed to the reform than a firm with 20 young employees out of a total of 50 employees even though the latter firm experiences a substantially larger labor cost reduction in monetary terms following the payroll tax cut.

We argue that the largest effect of the reform is likely to be for those firms that receive labor cost savings covering a large part, or even the whole cost, of any additional employees. We thus use the labor cost saving per firm at the time of the payroll tax cut as our treatment intensity measure. We construct five equally sized quantiles across the distribution of firms’ labor cost savings to investigate if the effect of the reform depends on the treatment intensity dose. A potential concern with our approach is that the size of labor cost savings and firm size are positively correlated, which induces the risk of differences in firm size biasing the estimate of the employment effect because large firms tend to grow more in absolute terms than small firms (Henrekson and Johansson 2010). To handle this potential source of bias, we rely on a firm-level difference-in-difference-in-differences (DDD) model. In contrast to ordinary difference-in-difference (DiD) estimation, the DDD model eliminates any underlying bias caused by differential trends in absolute employment growth between the treatment and control groups.

Our study contributes to a small but growing literature that takes into account firms’ labor cost savings of payroll tax reforms. Cottet (2020), for example, investigated a payroll tax cut for minimum wage workers in France, finding that firms with many minimum wage workers primarily increased their number of employees with higher wages. Ku et al. (2020) analyzed the employment effects when geographically based payroll taxes in Norway were suddenly abolished and found a significant reduction in local employment where firms received a payroll tax hike. When investigating a regional payroll tax reform in Finland, Benzarti and Haju (2020) found that firms who received a payroll tax cut became more resilient towards recessions compared to similar firms that were located in regions that did not receive any payroll tax cut. Overall, these studies seem to imply that employment effects of payroll tax reforms are stronger if they are targeted towards firms rather than individuals (see also Saez et al., 2019a, 2019b; Cottet, 2020).

We find that 80% of all treated firms saved less than 60,000 SEK annually ($6,480)^1^, suggesting that most firms experienced fairly modest decreases in their labor costs following the Swedish youth payroll tax cut in 2007. This finding might explain why most previous studies have found relatively small employment effects from the payroll tax reform. We do, however, observe a large variation in labor cost savings across firms, and we find that employers who received a large treatment intensity dose increased their number of employees significantly more than employers who received a low treatment intensity dose.

More specifically, we find that the average firm within the >0–20 % treatment intensity interval of the labor cost savings distribution recruited an additional 0.13 employees following the youth payroll tax cut. The corresponding figures for firms within the >60–80 % and >80–100 % treatment intensity ranges amount to 0.38 and 0.90 employees, respectively. We also find that the payroll tax cut mainly is associated with an increased number of employees within the targeted age group (19-25 years of age). However, we find some indications that employers also increased their recruitment of older individuals, implying that the immediate labor cost savings created by the reform also had a positive yet small employment effect outside the targeted age group.

We conclude that the employment effect of the 2007 Swedish payroll tax reduction is strongly contingent on the size of firms’ labor cost savings, and it is therefore primarily determined by the prereform age composition of firms’ personnel. In total, we estimate that the reform created 18,100 new jobs. We also find indications that the number of work hours increased among incumbent employees who are working at firms with small labor cost savings, while we find the opposite results for those working at firms that received large labor cost savings. Our interpretation is that nontargeted incumbents’ work tasks at firms receiving a high treatment intensity dose partly have been overtaken by those younger employees that were targeted by the reform.

The outline of this paper is as follows. The next section describes the 2007 Swedish payroll tax reform and provides main findings from previous evaluations. Section 3 includes a description of our data, and our treatment intensity measure, and presents descriptive statistics, while our empirical methodology is explained in Section 4. The empirical findings for how the reform affected firm-level employment and wages are presented in Section 5. Lastly, Section 6 summarizes our results and concludes the paper.

## The 2007 Swedish Payroll Tax Reform

The Swedish payroll tax is paid entirely by employers and is proportional to the gross wages of the employees. This tax has increased substantially during the last 50 years: from 11.65% in 1970 to 31.42% in 2019 (Swedish Tax Authority 2019). The payroll tax consists of seven different fees, all but one of which finance various social benefits such as pensions, parental leave, and sick leave.^2^

On July 1, 2007, the center-right government reduced the payroll tax for individuals who had turned 18 but not yet turned 25 at the start of the year. The aim of the reduction was to decrease the relatively high and growing unemployment rate among young individuals at that time (Swedish Government 2006). Of the seven different fees that jointly constituted the payroll tax, six fees were halved and the payroll tax was reduced from 32.42 to 21.32% for individuals within the targeted age group.^3^ The Swedish government argued that the reduction would result in a substantial amount of foregone tax revenues, which would be counterbalanced by an increased tax collection from labor incomes (Swedish Government 2006).

The reform was extended on January 1, 2009, by imposing a further reduction of the payroll tax rate to 15.49% and by widening the age group to all individuals who had not yet turned 26 by the start of 2009. Thus, the lower age bound was abolished and all individuals born in 1983 or later were targeted by the extension in 2009 (Swedish Government 2008).

The political left-wing parties, which were in opposition at the time, criticized the reform. They argued that the reform was inefficient and costly considering the size of foregone tax revenues. Once elected into office in 2014, the left-wing government decided to restore the payroll tax level for young individuals to 31.42%. The payroll tax reduction for young employees was completely abolished on June 1, 2016.

A number of studies have previously investigated the labor market effects of the youth payroll tax cut in 2007. Egebark and Kaunitz (2013) used a difference-in-difference model to compare the employment outcomes for young individuals who were targeted by the reform with the outcomes for slightly older individuals who were noneligible for the payroll tax reduction. They found that the reform increased the employment of young individuals by 2.7 percentage points, and that the payroll tax cut had only minor effects on their wages. Overall, they estimated the 2007 reform to have created between 6,000–10,000 new jobs per year within the targeted age group.

In a closely related paper, Egebark and Kaunitz (2014) investigated the long-term effect of the reform and whether the reduction increased the number of hours worked among those already employed. They found no indications of employment increases along the intensive margin, while the employment effect within the target group declined with age along the extensive margin. Hence, the reform appeared to have been most beneficial for the youngest employees, i.e., those who were eligible for the longest period.

From these two studies, Egebark and Kaunitz (2013, 2014) concluded that the Swedish payroll tax reduction was largely unsuccessful in decreasing youth unemployment. They highlighted that productivity among many youths might have remained too low in relation to the reduction in their labor costs succeeding the reform. Additionally, they also considered the reform to be costly as the foregone payroll tax revenues per created job was estimated to be 1.2 million SEK.

Skedinger (2014) analyzed the effects of the payroll tax reform on employment within the retail industry, which is an industry with a high proportion of young employees. Skedinger found only small positive effects of the payroll tax cut on young individuals’ employment. However, the estimated effect was larger for employees who had a wage close to the negotiated minimum wage, which indicated that the relatively high entry-wages within the retail industry might obstruct the entry of young individuals into the labor market. At the firm-level, Skedinger also evaluated whether a higher prereform share of young employees, i.e., treatment intensity, was associated with improved firm performance and found some evidence of an increased profit margin. These findings were, however, derived from a sample of only 354 firms, suggesting a potentially limited external validity.

Two recent studies that have acknowledged the link between the number of young employees and the magnitude of firms’ labor cost savings are Egebark and Kaunitz (2017) and Saez et al. (2019a). The former study used the 2006 firm-level wage bill for the targeted group, normalized by firm turnover, as a proxy for how sensitive firms’ labor costs were to the payroll tax reduction, i.e., as a treatment intensity measure. Next, they studied the link between firms’ treatment intensity and their change in performance in terms of profits, labor productivity and investments. They found no evidence suggesting that a higher treatment intensity was associated with improved firm performance.

Saez et al. (2019a) constitutes the research that is most in line with our study. They analyzed the payroll tax reform and its implications from both an individual-level and a firm-level perspective. By tracing different cohorts of individuals over time, they assessed how employment rates and wages within different age groups changed when the payroll tax cut was implemented. For individuals within the targeted age group, they found noticeable increases in employment, but they also found that net wages were unaffected, implying that the employment effects were not offset by potential wage spillovers.

In addition, Saez et al. (2019a) used the 2006 firm-level share of total wage costs spent on young employees as a proxy for firms’ exposure to the reform. Their treatment group consisted of firms within the top quartile of the distribution, while their control group included firms within the middle-50% of the distribution (medium share). To further exploit the variation in treatment intensity, they split their treatment group into two groups; these groups are referred to as the fairly high and very high share groups, respectively. Next, they used difference-in-difference estimation to compare outcomes among firms with high and low treatment intensities and found employment increases of 2.8–6.5% over the 2003–2013 period, varying with firms’ exposure to the reform. In addition, they found that the reform resulted in wage increases for older incumbent workers who were not targeted. Saez et al. (2019a) were not able to estimate the total number of jobs created by the payroll tax cut since their control group consisted of firms that also received labor cost savings following the reform.

## Data, Treatment Intensities, and Descriptive Statistics

### Data

Our analysis is based on LISA (Longitudinal Integration Database for Health Insurance and Labor Market Studies), a register-based database provided by Statistics Sweden. LISA covers all Swedish residents that are least 16 years old and provides data on, for example, individuals’ employment status, educational background and annual earnings.

In LISA, we can observe the yearly employment status of each individual during the month of November. This information is collected from the RAMS (Labor Statistics Based on Administrative Sources) register, where an individual is classified as employed if (s)he has a labor income corresponding to at least one work hour during a specific measurement week in November. This definition coincides with the one used by the International Labor Organization (ILO), but it captures a very heterogeneous sample of individuals, including both part-time and full-time workers. In line with Mörk et al. (2014), we therefore also utilize two more restrictive income-based employment definitions as a robustness check. These definitions require, in addition to being registered as employed in RAMS, an individual to have had annual labor earnings of at least one- or two-income base amounts, respectively.^4^

Employment and unemployment statuses in LISA are furthermore based on two different registers and measured at different time points in November, which means that a limited a number of individuals are simultaneously registered as employed and unemployed in the dataset. We choose to define these employees as unemployed and exclude them from our analysis since we cannot with any certainty conclude that they are regularly employed.

LISA includes a unique firm identification number, making it possible for us to connect each employee with his/her employer during the month of November. Each employee is assigned an industry code from the SNI2002 (Swedish Standard Industrial Classification) system, which consists of 776 industries at the most detailed (five-digit) level and 60 industries at the most aggregated (two-digit) level. For each firm, we assign the most frequent industry code. The data includes the total gross yearly wage that each employee received from his/her primary employer. We utilize this information in combination with the age of the individuals to calculate the gross wages paid to different age groups by each employer.^5^ By connecting employees and their employers, we obtain a matched employer-employee dataset of Swedish firms from 2003 to 2008. In total, our dataset contains information on 744,032 firms.

To ensure that the empirical analysis is not affected by firm outliers, we exclude all firms that had extreme yearly changes in employment.^6^ Our outlier definition applies for 1539 firms, leaving us with 742,493 firms. Moreover, we exclude 64,422 firms that are assigned the industry code 0, meaning that we end up with 678,071 firms over the 2003–2008 period.^7^ Our regression analyses are built upon two separate periods: 2003–2005 and 2006–2008. In these analyses, we exclude firm entries and exits and therefore only analyze surviving firms with at least one employee per year over the respective time period. Consequently, our firm panel includes 265,839 firms over 2003–2005 and 295,771 firms over 2006–2008. For the firms to be included in the empirical analyses, they must additionally have nonmissing values for total wages for young people; control firms are also required to have not had any young employees in 2006 in addition to having a youth wage sum equal to zero.

### Treatment Intensities

The 2007 Swedish payroll tax reform reduced labor costs for all firms that had young employees at the time of the reform. Following Saez et al. (2019), we consider firms to have received different doses of the payroll tax cut, i.e., to have different treatment intensities that depend on the size of their labor cost savings. However, since our focus is on the overall employment effect of the reform (number of employees), and because we want a treatment measure that is strictly increasing in labor cost savings due to the reform, we consider firms’ exposure to the reform in terms of the absolute size of their labor cost reductions.

**Fig. 1:**
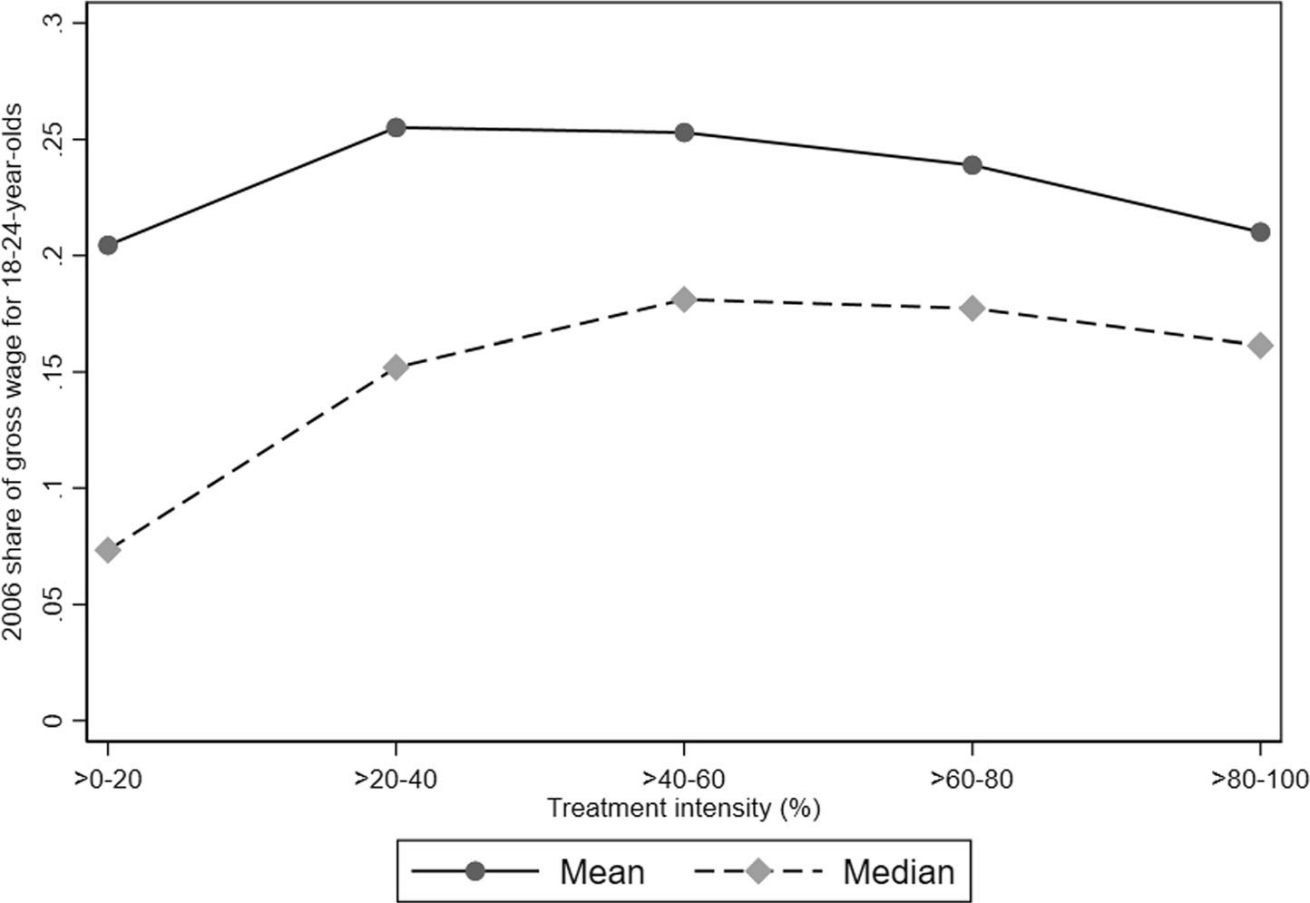
Relative treatment intensity by the absolute size of labor cost savings. Note: The graph illustrates firms’ average and median relative treatment intensities according to their absolute treatment intensity group. The relative treatment intensity is represented by the share of 2006 gross wages spent on 18–24-year-olds (vertical axis). Surviving firms with at least one employee over 2006–2008 are included. Outliers (defined as annual employment changes of more than three standard deviations from average change (+/- 88 employees)) are excluded

To clarify why this distinction is important, Fig. 1 depicts the relationship between the Saez et al. (2019a) relative measure (vertical axis) and our absolute measure (horizontal axis). More specifically, Fig. 1 plots the 2006 average and median firm-level share of total gross wages spent on 18–24-year-olds by our 2006 absolute treatment intensity measure. 8

Figure 1 shows that there is no strong link between a firms’ relative and absolute treatment intensity. The relative treatment intensity is, on average, very similar for firms with the largest (>80–100 group) and smallest (>0–20) absolute labor cost savings, which implies that how much a firm saves in absolute terms is unrelated to the share of total wages spent on young employees. We find it likely that managers in most firms will use the labor cost savings in SEK (perhaps relative to the wage of the potential new employee) in their evaluation of whether to hire or not, rather than using the savings as a share of total wages. If this is the case, firms will tend to recruit new employees based on how much they save in absolute monetary terms, and not on their relative cost savings.

As such, we use a treatment intensity measure that strictly increases with the size of firms’ labor cost savings. More specifically the 2006 Treatment intensity_*i*, *t*_ of firm *i* at year *t* can be written as:

$$ {\mathrm{Treatment}\ \mathrm{intensity}}_{i,t=2006}=\left(0.3242-0.2132\right)\times W\_{\mathrm{young}}_{it=2006} $$

where the figures 0.3242 and 0.2132 represent the payroll tax levels before and after the tax cut, respectively; and *W* _ young_*it*_ represents the total gross wages (excluding payroll taxes) payed to individuals covered by the tax reform.

Our treatment intensity measure is constructed as follows. First, we calculate the total gross wages paid by each firm to their employees aged 18–24 in 2006, i.e., those employees who will be eligible in 2007. Since the payroll tax cut was implemented in mid-2007, this measure works as a proxy for expected labor cost savings from mid-2007 to mid-2008. We expect a strong correlation between our estimated labor cost reductions and the actual reductions that occurred at the time of the reform.^9^ Importantly, by defining the treatment intensity in 2006, we ensure that it is predetermined, i.e., that firms’ treatment intensities are unaffected by their hiring post-reform. We then multiply *W* _ young_*it*_ by 0.111, which is the percentage reduction in payroll taxes for these employees once the reform is introduced. In turn, this implies that Treatment intensity_*i*, *t* = 2006_ measures the size of the one-year labor cost savings that firms receive for having young employees in 2006 provided that they remain employed in 2007 at the same wage levels.

Next, we split all firms satisfying Treatment intensity_*i*, *t* = 2006_ > 0 into five equally sized quantiles based on their rank in the treatment intensity distribution. The lowest quantile includes the firms with the smallest 20% of labor cost savings; the highest quantile includes the firms with labor cost savings in the top >80–100% of the treatment intensity distribution. Our control group includes all firms that lacked employees aged 18–24 in 2006 and therefore did not obtain any immediate labor cost savings due to the payroll tax reform. The control group thus differs from that used by Saez et al. (2019a), wherein the control consisted of firms that were also exposed to the reform. Our control group enables us to estimate the total employment effect of the payroll tax reform and not only how the employment effect varies with the size of firms’ labor cost savings.

**Fig. 2:**
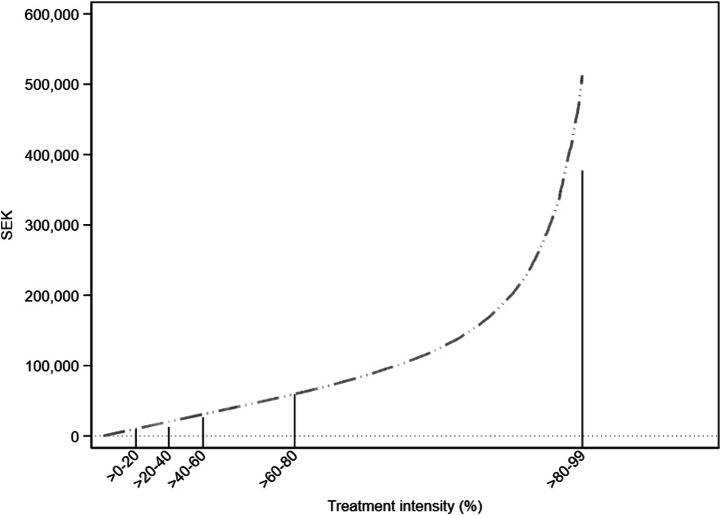
Labor cost savings by continuous treatment intensity. Note: The graph shows the expected one-year labor cost savings measured in SEK on the y-axis. The continuous treatment intensity rate is presented on the x-axis. Includes surviving firms with at least one employee per year over 2006–2008. Outliers (defined as annual employment changes of more than three standard deviations from average change (+/- 88 employees)) are excluded. Since some firms have savings in excess of 5 million SEK, which would make the graph difficult to visualize if included, we exclude savings above the 99th percentile from the graph. Measured according to the price level of 2016. 1 SEK = 0.108 USD

The relationship between labor cost savings and treatment intensities, with the continuous treatment intensity rate on the x-axis, is presented in Fig. 2. Note that of all treated firms, i.e., firms with Treatment intensity_*i*, *t* = 2006_ > 0, 80% saved less than 60,000 SEK (6,480 USD) during the first year after the youth payroll tax cut was implemented, which is far less than the average annual labor cost of a full-time employee in Sweden. Thus, a vast majority of employers received relatively small labor cost savings due to the reform, which might explain why previous studies have reported small employment effects. We do, however, notice a substantial variation in cost savings within the >80–100 group, with the 99th treatment intensity percentile corresponding to savings of approximately 513,000 SEK (55,404 USD).

The range of the labor cost savings for each treatment intensity group is presented in Table 1, including the 100th percentile for the >80–100 group. Here, we can notice a maximum 1-year saving amounting to almost 5.4 million SEK (583,200 USD).

**Table 1:** Labor cost savings by treatment intensity group

Dose group	2006 savings in SEK	Mean	Median
>0–20%	124–10,205	6060	6165
>20–40%	10,217–20,310	15,075	14,986
>40–60%	20,323–30,812	25,352	25,277
>60–80%	30,824–59,634	42,168	40,547
>80–100%	59,647–5,391,096	172,697	103,782

Note: Includes surviving firms with at least one employee per year over 2006–2008. Outliers (defined as annual employment changes of more than three standard deviations from average change (+/- 88 employees)) are excluded. Measured according to the price level of 2016. 1 SEK = 0.108 USD

### Descriptive Statistics

Table 2 presents descriptive statistics for our treatment and control groups in 2006. The average firm size is noticeably larger in the treatment groups than in our control group. Both the average and median firm size increase with treatment intensity, which is because large labor cost savings in absolute terms are associated with a large number of employees. For instance, the average firm in the highest dose group has approximately 74 employees, whereas the average firm in the control group has fewer than three employees. Note also that a vast majority of employees are older than 25. Turning to the share of young employees (18–24-year-olds in 2006), between approximately one fourth and one third of the individuals at the treated firms were soon to be covered by the reduced payroll tax. Finally, we note that each dose group contains nearly 11,000 firms. In total, we analyze the employment changes for approximately 54,000 treated firms and 222,000 control firms over the period of 2006–2008.

**Table 2:** Descriptive statistics for the treatment and control groups, 2006

	Mean	Median	Std.dev.	Min	Max	# Firms
Firm size (# employees)						
Control	2.545	1	5.801	1	490	221,976
Dose >0–20%	8.545	4	18.906	1	680	10,878
Dose >20–40%	9.065	5	17.510	1	606	10,864
Dose >40–60%	10.950	6	21.449	1	658	10,854
Dose >60–80%	16.802	10	28.098	1	702	10,878
Dose >80–100%	73.751	30	163.782	1	2,829	10,866
# 19-25 yrs old						
Control	0.030	0	0.190	0	9	221,976
Dose >0–20%	1.197	1	1.023	0	33	10,878
Dose >20–40%	1.512	1	1.084	0	29	10,864
Dose >40–60%	1.687	1	1.222	0	20	10,854
Dose >60–80%	2.798	2	1.818	0	25	10,878
Dose >80–100%	11.846	7	18.008	1	450	10,866
# >25 yrs old						
Control	2.510	1	5.753	0	488	221,976
Dose >0–20%	7.122	3	18.630	0	679	10,878
Dose >20–40%	7.417	3	17.121	0	599	10,864
Dose >40–60%	9.128	4	20.100	0	649	10,854
Dose >60–80%	13.744	7	27.470	0	677	10,878
Dose >80–100%	60.846	21	150.982	0	2,536	10,866
Share of young						
Control	0	0	0	0	0	221,976
Dose >0–20%	0.338	0.25	0.269	0.001	1	10,878
Dose >20–40%	0.334	0.25	0.258	0.003	1	10.864
Dose >40–60%	0.295	0.25	0.230	0.004	1	10,854
Dose >60–80%	0.293	0.25	0.213	0.003	1	10,878
Dose >80–100%	0.279	0.234	0.188	0.004	1	10,866

Note: Includes surviving firms with at least one employee per year over 2006–2008. Outliers (defined as annual employment changes of more than three standard deviations from average change (+/- 88 employees)) are excluded

## Empirical Method

Firms’ labor cost savings generated by the payroll tax reform strictly increase with our treatment intensity measure. However, the amount of labor cost savings and firm size are positively correlated, which means that the average firm size also increases with our treatment intensity measure. Previous research has shown that larger firms grow more than small firms in absolute terms (Henrekson and Johansson 2010), and it is thus likely that firms within the treatment groups would have experienced higher employment growth than the control group firms even in the absence of the youth payroll tax reform. To correctly identify the treatment effect, we must therefore ensure that the treated and control group firms would have had similar employment growth patterns in absence of the reform, i.e., that the control firms resemble the counterfactual employment outcome of the treated firms.

We rely on a firm-level difference-in-difference-in-differences (DDD) model (Chetty et al. 2009; Gruber 1994) to achieve this goal. The DDD model compares the employment among treated and control firms before and after treatment for two different time periods. In contrast to a difference-in-difference (DiD) model, the DDD model eliminates bias by deducting potential differences in employment growth trends between the treated and control firms during the prereform time period of 2003–2005.

**Fig. 3:**
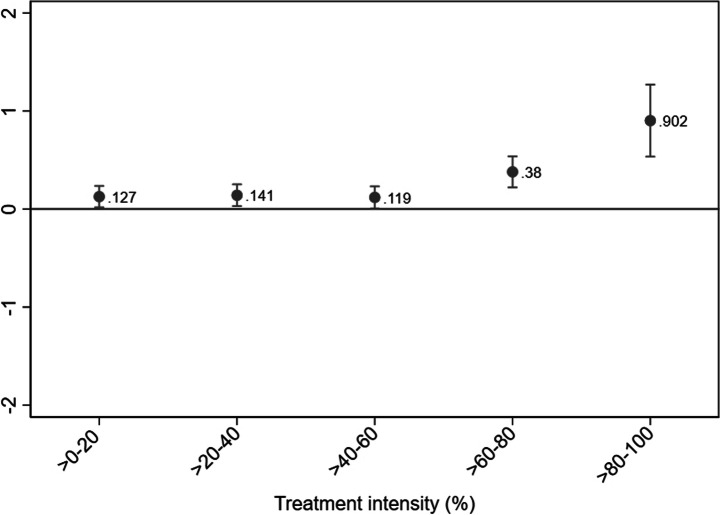
Employment effects by treatment intensity. DDD estimation. Notes. Dependent variable: Firm size (number of employees). Treatment period: 2006–2008. Underlying time period: 2003–2005. Within-firm estimation. Only surviving firms with at least one employee per year are included. Outliers (defined as annual employment changes of more than three standard deviations from average change (+/- 88 employees)) are excluded. Firm clustered standard errors. Point estimates with 95% confidence intervals

More specifically, the DDD model captures the difference between two DiD estimates across the time periods of 2003–2005 and 2006–2008, respectively.^10^ The underlying DiD for the time period 2003–2005 accounts for differences in employment growth between treatment and control firms in the prereform period. By deducting this estimate, we ensure that the estimated employment effects are not affected by nonparallel trends in employment growth between the treated and control firms in the pretreatment period.^11^ Note that if no differences in employment growth exist during 2003–2005, our DDD model and an ordinary DiD model for the period 2006–2008 would provide identical estimates. For clarification, we thus also decompose the estimates of Fig. 3 below into separate DiD estimates over the time periods of 2003–2005 and 2006–2008, respectively.^12^

Our DDD model can be expressed as:

1 $$ {\displaystyle \begin{array}{c}{Size}_{ijt}=\alpha +{\beta}_1{ Tim e}_t+{\beta}_2{Group}_j+{\beta}_3{Treat}_i+{\beta}_4\left({Group}_j\ast {Tim e}_t\right)+{\beta}_5\left({Treat}_i\ast {Tim e}_t\right)+{\beta}_6\left({Group}_j\ast {Treat}_i\right)\\ {}+{\delta}_{DDD}\left({Group}_j\ast {Treat}_i\ast {Tim\mathrm{e}}_t\right)+{\varepsilon}_{ijt}\end{array}} $$

where *i* denotes firm, *j* denotes group (treated or control) and *t* denotes year. *Time*_*t*_ is a time indicator that is equal to zero for the years 2003 and 2006 and equal to one for the years 2004, 2005, 2007, and 2008. Thus, *Time*_*t*_ is equal to one for the post-treatment years of both the actual reform period 2006-2008 and the prereform period 2003-2005 used to account for underlying differences in employment trends between treated and control firms; *Group*_*j*_ is a group indicator equal to zero for the control groups used in the prereform time period 2003–2005 and the reform period 2006-2008 and equal to one for the corresponding treatment groups; and, finally, *Treat*_*i*_ separates all firms used in the prereform period of 2003–2005, i.e., that account for differential employment trends, from firms analyzed in the reform period of 2006-2008 by being equal to zero for the former group and equal to one for the latter group.

Our main variable of interest is the interaction term of these three variables: *Group*_*j*_ ∗ *Treat*_*i*_ ∗ *Time*_*t*_. This variable takes on the value of one for the treatment group in the post-treatment years of the actual reform period, i.e., 2007-2008. The population parameter, *δ*_*DDD*_, of the variable captures the treatment effect of reduced payroll taxes on employment, and is net of other factors that could cause differences in employment growth between treated and control firms. *δ*_*DDD*_ can be written as:

$$ {\displaystyle \begin{array}{c}{\delta}_{DDD}=E\left[{Size}_{ijt}|{Time}_t=1,{Group}_j=1,{Treat}_i=1\right]\\ {}-E\left[{Size}_{ijt}|{Time}_t=0,{Group}_j=1,{Treat}_i=1\right]\\ {}\begin{array}{c}-\left(E\left[{Size}_{ijt}|{Time}_t=1,{Group}_j=0,{Treat}_i=1\right]\right.\\ {}\left.-E\left[{Size}_{ijt}|{Time}_t=0,{Group}_j=0,{Treat}_i=1\right]\right)\\ {}\begin{array}{c}-\left(E\left[{Size}_{ijt}|{Time}_t=1,{Group}_j=1,{Treat}_i=0\right]\right.\\ {}-E\left[{Size}_{ijt}|{Time}_t=0,{Group}_j=1,{Treat}_i=1\right]\Big)\\ {}\begin{array}{c}-\Big(E\left[{Size}_{ijt}|{Time}_t=1,{Group}_j=0,{Treat}_i=0\right]\\ {}-E\left[{Size}_{ijt}|{Time}_t=0,{Group}_j=0,{Treat}_i=0\right]\Big)\end{array}\end{array}\end{array}\end{array}} $$

where the first four lines of the expression represent a regular difference-in-difference estimate across the reform period of 2006-2008, while the last four lines represent an underlying difference-in-difference estimate across the prereform period of 2003–2005, which accounts for initial differences in employment growth. Hence, our DDD model generates the employment effect of the payroll tax reform by deducting potential bias caused by the treatment and control groups having different pretreatment trends in employment.^13^

We estimate our DDD model separately for each treatment intensity group. Hence, the obtained parameter estimates $\hat{\delta}$ will indicate if the employment effect varies with the size of labor cost savings, which is to be expected if it is the absolute size of labor cost savings that causes firms to hire.

## Findings

### Effects on Number of Employees

From a theoretical firm-level perspective, we should expect reduced labor costs for young employees to give rise to both a substitution effect and a scale (output) effect.^14^ When one input factor, namely young employees, becomes less costly, the substitution effect incentivizes the firm to rearrange its mix of input factors and shift towards a more youth labor-intensive production. However, the labor cost savings due to the youth payroll tax cut also causes a downward shift in the marginal cost curve, i.e., producing any given level of output becomes cheaper. This downward shift creates a scale effect that incentivizes an expanded production and, thus, an increased usage of other input factors as well.^15^ Due to this scale effect, a firm might also increase its employment of individuals who are not directly targeted by the reduced payroll tax.^16^ Importantly, the marginal cost of production decreases more for a firm with large labor cost savings than for a firm with small labor cost savings. The magnitude of both the substitution and scale effects is therefore positively related to the size of labor cost savings, suggesting that that we, from a theoretical standpoint, could expect the largest employment increases for both young (eligible) and older (noneligible) individuals among firms with the highest treatment intensities.

For our baseline results, we estimate model (1) using OLS within-firm estimation. Consequently, we account for any time-invariant firm-specific effects that might affect our results. In the Appendix, we provide alternative specifications in which we account for heterogeneity across both industries and municipalities (see Table A2–A4).^17^ All employment effects are estimated over the 2006-2008 period, meaning that we estimate the employment effects during the first 18 months after the reform introduction. The estimated employment effects for all individuals (irrespective of age) are presented in Fig. 3 together with their associated 95% confidence intervals.^18^

We find that the reform is associated with statistically significant employment increases in all of the five treatment intensity groups. Within the >0–20 treatment intensity range, the average increase amounts to 0.13 employees. The corresponding employment effects within the >60–80 and >80–100 ranges amount to 0.38 and 0.90 employees, respectively. The magnitude of the point estimates thus increases with treatment intensity, suggesting a positive link between the size of firms’ labor cost savings and their subsequent employment. The confidence interval for the >80–100 group also shows that those firms that received the largest labor cost savings increased their number of employees by significantly more than firms within the >0–60 treatment intensity range.

By multiplying the average employment increases over 2006–2008 by the corresponding number of firms (Table 2), we compute the estimated number of jobs created. We find the 2007 payroll tax cut to be associated with 18,100 jobs, of which nearly 10,000 were created by firms within the top-20 treatment intensity range.^19^

One potential concern is that the large employment effect found within the >80–100 group might be driven by only a few firms with substantial labor cost savings, which would imply that the point estimate is not representative for firms in general within that group. To investigate whether this is the case, we further split the >80–100 firms into the treatment intensity ranges >80–85, >85–90, >90–95, and >95–100%. We find that each firm within the >85–90 range hired on average 0.68 employees, whereas the average effect within the >90–95 and >95–100 groups amounts to 1.35 and 1.44 employees, respectively (see Figure A3 in the Appendix). This finding illustrates that our results are not solely driven by a small number of firms belonging to the top 5% of the savings distribution.

For reasons of comparison, we also estimate the employment effects using a traditional difference-in-difference model (DiD). As mentioned, our DDD model estimates the difference between two DiD models across the time periods of 2006–2008 and 2003–2005, respectively. Thus, the treated and control firms had different employment trends prior to the payroll tax reform if the 2006–2008 estimates using DDD and DiD differ. Conversely, similar 2006–2008 estimates from DDD and DiD would imply parallel pretreatment trends and, hence, that the underlying DiD for the time period of 2003–2005 renders insignificant estimates. The DiD estimates can be found in the Appendix (see Figure A1).

Estimating the 2006–2008 employment effects using DiD generally yields substantially larger point estimates than using DDD. For instance, the estimated employment effect within the >80–100 range amounts to 1.23 individuals, which should be compared to the corresponding estimate of 0.90 in Fig. 3, above. Furthermore, the confidence intervals are typically narrower using DiD, yielding higher statistical significance. Since the DiD estimates are considerably larger than the DDD estimates, it implies that the treated firms had a higher employment growth than the control firms in the prereform years of 2003–2005. Evaluating the DiD estimates for the 2003–2005 period reveals that this is the case for some of the treatment intensity groups (lower part of Figure A1). This finding further motivates our choice to rely on DDD to accurately estimate employment effects.

Next, we investigate the effect of the payroll tax reform on the recruitment of employees within the targeted age group (19–25-year-olds) and compare it with the recruitment effect of older individuals (above 25 years of age). This comparison is important because the treated firms, on the one hand, may have recruited more young employees after the reform as a direct consequence of the 11-percentage point reduction in the payroll tax for young individuals. As discussed at the beginning of this section, this would reflect a substitution effect. On the other hand, the labor cost savings also reduced firms’ marginal cost of production, which could have incentivized the firms to recruit more experienced and senior employees.

**Fig. 4:**
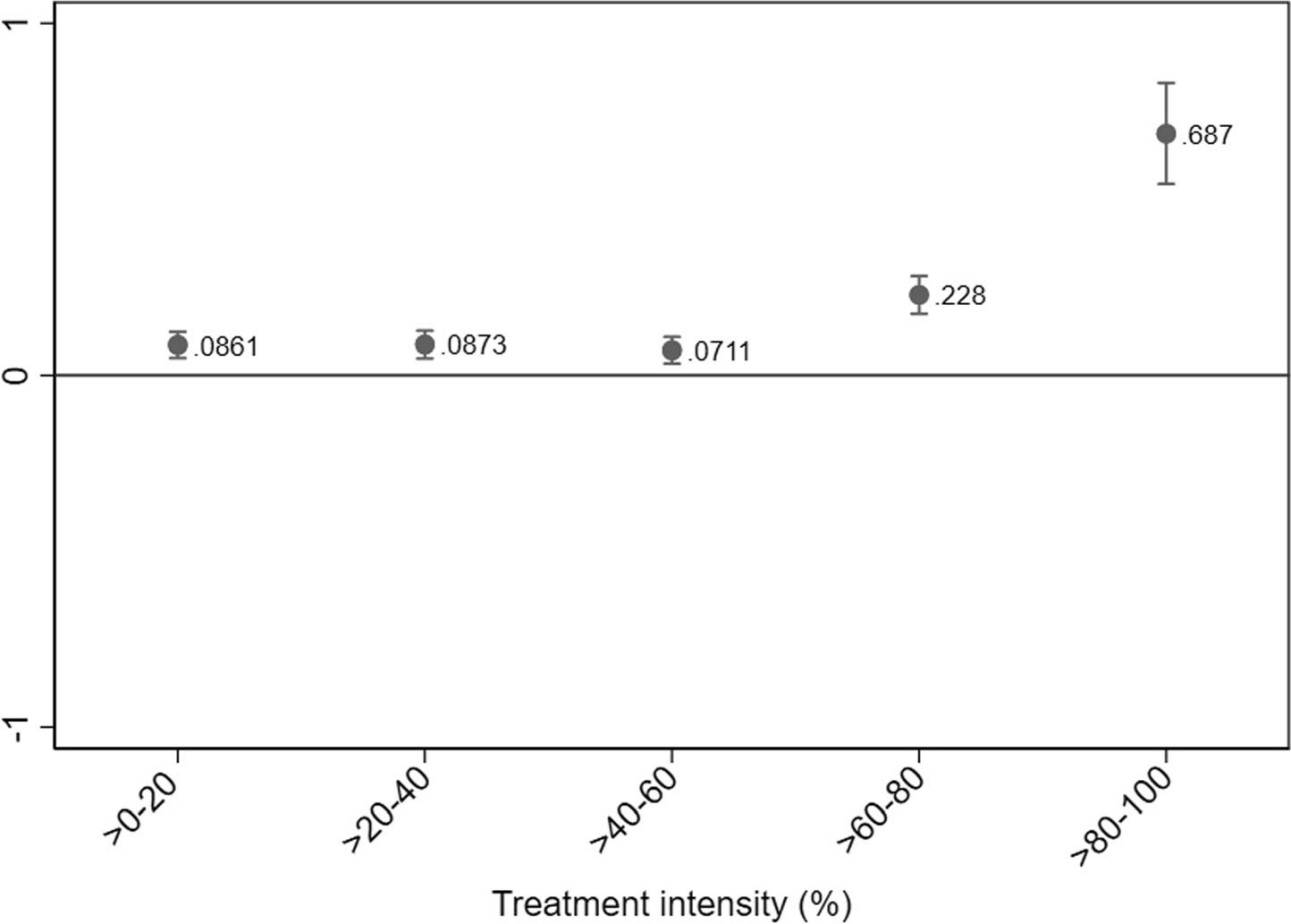
Employment effect among 19–25-year-olds by treatment intensity. DDD estimation. Notes. Dependent variable: Number of young employees aged 19–25. Treatment period: 2006–2008. Underlying time period: 2003–2005. Within-firm estimation. Only surviving firms with at least one employee per year are included. Outliers (defined as annual employment changes of more than three standard deviations from average change (+/- 88 employees)) are excluded. Firm clustered standard errors. Point estimates with 95 % confidence intervals

The estimated employment effects for 19–25-year-olds are presented in Fig. 4. We obtain statistically significant point estimates for all treatment intensity groups, suggesting employment increases of the young employees targeted by the reform. Moreover, we again find a positive link between the firms’ labor cost savings and their subsequent employment change. For firms in the highest treatment intensity group, we find the payroll tax cut to have increased the employment of individuals within the targeted age group by, on average, 0.69 employees per firm. Relating the point estimates to the corresponding number of firms to compute the estimated number of jobs created, we conclude that approximately 12,600 of the 18,100 jobs in total were created for the young individuals targeted by the reform.

**Fig. 5:**
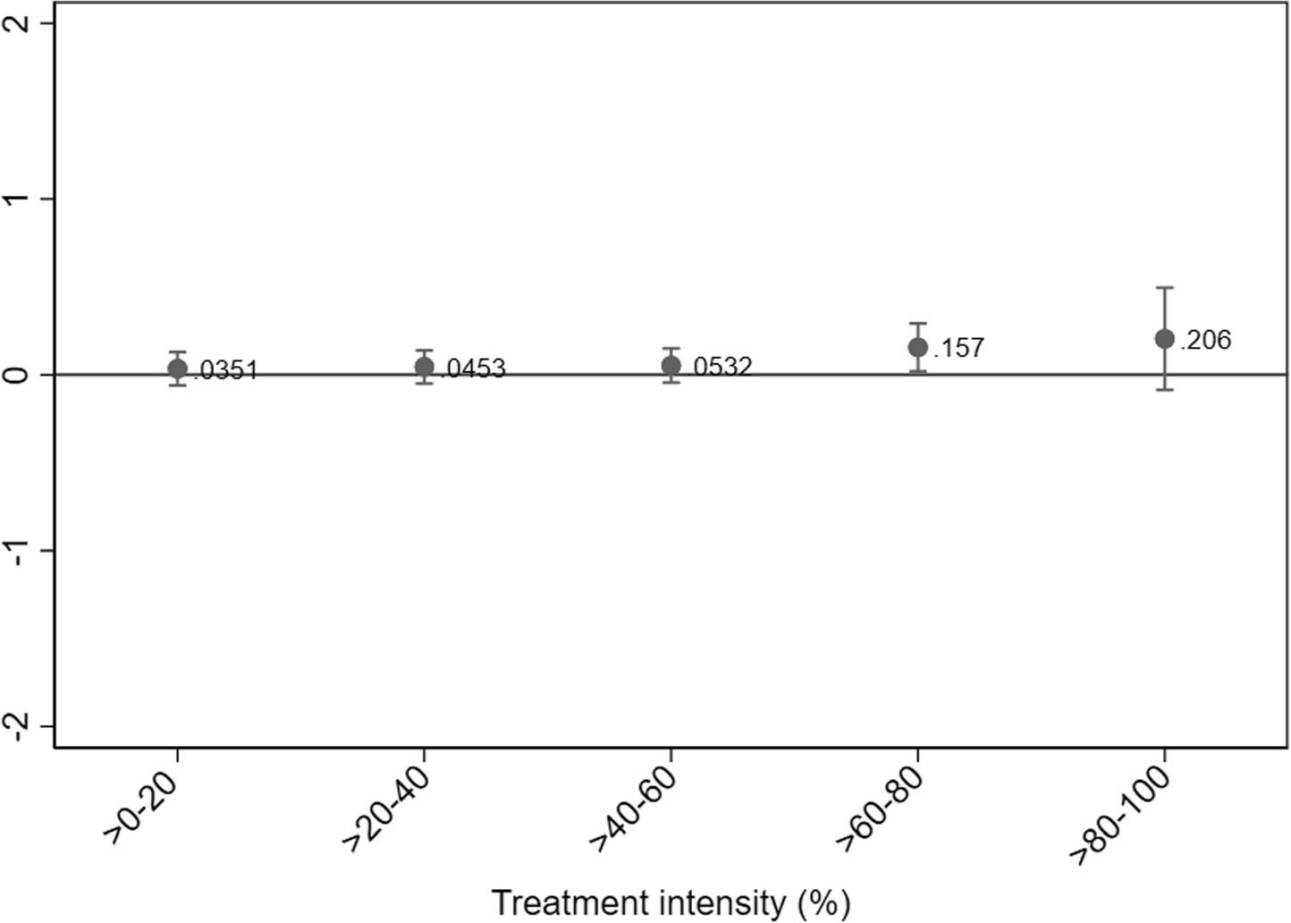
Employment effect among older individuals by treatment intensity. DDD estimation. Notes. Dependent variable: Number of employees above the age of 25. Treatment period: 2006–2008. Underlying time period: 2003–2005. Within-firm estimation. Only surviving firms with at least one employee per year are included. Outliers (defined as annual employment changes of more than three standard deviations from average change (+/- 88 employees) are excluded. Firm clustered standard errors. Point estimates with 95 % confidence intervals

The estimated employment effects for individuals who were at least 26 years old at the time of the reform, i.e., those who were not directly targeted by the tax cut, are presented in Fig. 5. All but one of the DDD estimates are statistically insignificant. The exception is found in the second highest treatment intensity group, in which the payroll tax reform increased the employment of older individuals by, on average, 0.16 employees per firm. Note also that the point estimate for the largest treatment intensity group is positive and fairly similar in magnitude, but it is not statistically significant at the conventional 5% significance level.

To summarize, we find that the labor cost savings associated with the payroll tax reform had employment-promoting effects, and this held especially true among the firms with the largest savings. The positive employment effects are mainly explained by an increased recruitment of the reform’s target group, i.e., 19–25-year-olds, which reflects that the youth payroll tax cut gave rise to a substitution effect, incentivizing youth employment. We also find some indications of increased employment outside the targeted age group, which reflects a scale effect resulting from a reduction in firms’ marginal cost of production.

Our results are based on an employment definition that considers all employees that have worked at least one hour during a measurement week in November as employed. As a robustness check we have also included stricter income-based employment definitions. These definitions yield estimates that are larger—rather than smaller—in magnitude, suggesting that the employment growth among individuals having earnings of at least one income base amount has been larger than the general employment growth. Most jobs created by the payroll tax reform were thus provided for individuals who received steady labor incomes. These findings are omitted from the paper but are available from the authors upon request.

### Effects on Wages

In this section, we evaluate the extent to which the reduced labor costs were translated into increased wages. From a theoretical standpoint, an inelastic labor demand and labor supply leads to larger effects on wages and smaller effects on employment. In addition, the design of the wage bargaining system and the relative bargaining power of employers versus trade unions will determine the wage spillover effect (Bauer and Riphahn 2002; Bennmarker et al. 2009).

We focus our analysis on how the total wage sum for incumbent employees changed post-reform within the different treatment intensity groups. By limiting the analysis to incumbent employees, we ensure that our estimates are unaffected by firms’ employment decisions, which is important because the payroll tax cut particularly increased the employment of young individuals, who have below-average wages, indicating that our estimates could be underestimated if we were to include newly recruited individuals in the analysis.

Since we utilize the prereform period of 2003–2005 in our DDD model, our results presented below are based upon individuals who were working for the same firm during either 2003–2005 or 2006–2008. We limit the wage analysis to older employees who were not covered by the reduced payroll tax. The reason is that the control group, by definition, lacks young employees in 2006 and who are, thereby, incumbent over the reform period 2006–2008. Importantly, this does also allow us to evaluate if there were wage spillovers among noneligible individuals.

**Fig. 6:**
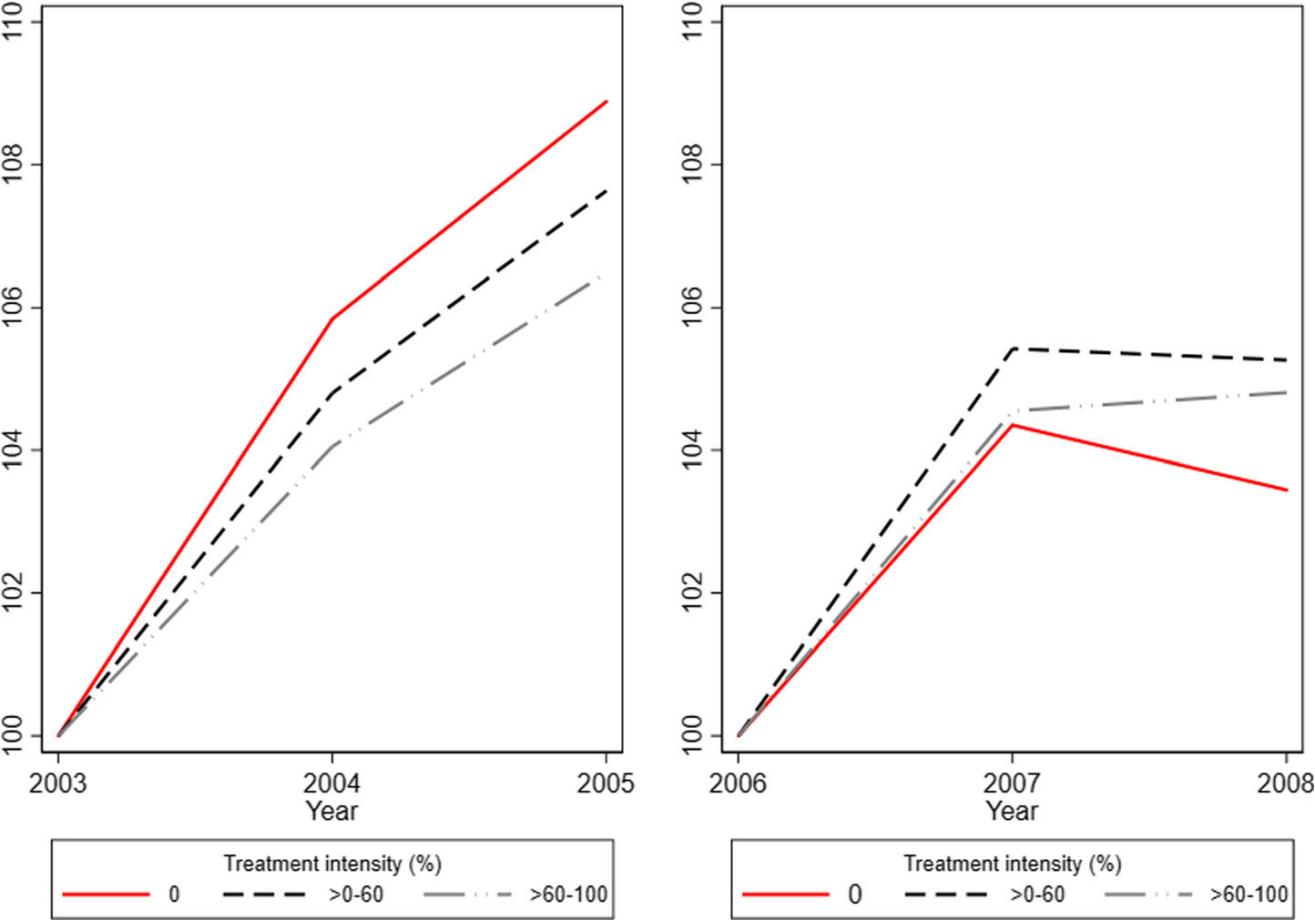
Percentage change of the average total gross wage sum for incumbent employees. Individuals older than 25 staying at the same firm during 2003–2005 or 2006–2008. Normalized to 2003 and 2006, respectively. Notes: Percentage change in the average gross wage sum for incumbent employees with a minimum age of 26. Base years: 2003 and 2006. Average wages are adjusted for inflation and measured according to the price level of year 2016. Outliers (defined as extreme annual changes in employment and/or wages) are excluded. Employment outliers are defined as annual employment changes of more than three standard deviations from average change (+/- 88 employees). Wage outliers are defined as annual changes in the average gross wage sum for incumbent employees of more than three standard deviations from the average change

We begin with a descriptive analysis in Fig. 6, which shows the percentage growth in the average wage sum of incumbent employees in the prereform period 2003–2005 and the reform period of 2006–2008. It thus compares the wage development of older (noneligible) incumbent employees over these two time periods and therefore provides a first indication of whether the wages were affected by the payroll tax reform. The treated firms are divided into two (rather than five) treatment intensity groups to make the figure easier to interpret. The first group merges all treated firms within the >0–60 range (the three lower quintiles) while the second group covers firms within the >60–100 range (the two upper quintiles). In both time periods, the average wage sum is normalized to the corresponding starting year.

Figure 6 shows that the wage trends are fairly similar between the (placebo) treatment and control groups in the prereform years of 2003–2005. Both groups experienced relative increases in the average wage sum of incumbent workers of between approximately 6.5 to 9%. Comparing the average wage development in the prereform years 2003–2004 and the reform years 2006–2007, we find that the percentage wage growth has been somewhat larger for the treated firms in the latter period, possibly implying that some of the labor cost savings generated by the reform was translated into wage increases for incumbent employees, i.e., employees who were already employed by the time of the reform introduction. However, when considering the entire time periods of 2003–2005 and 2006–2008, it is noticeable that the relative (percentage) wage growth was larger in the former period and that this holds for all groups. Additionally, there does not appear to be a positive link between the size of labor cost savings and wage increases since firms within the lower >0–60 group experienced a larger percentage increase than firms within the >60–100 group.

**Fig. 7:**
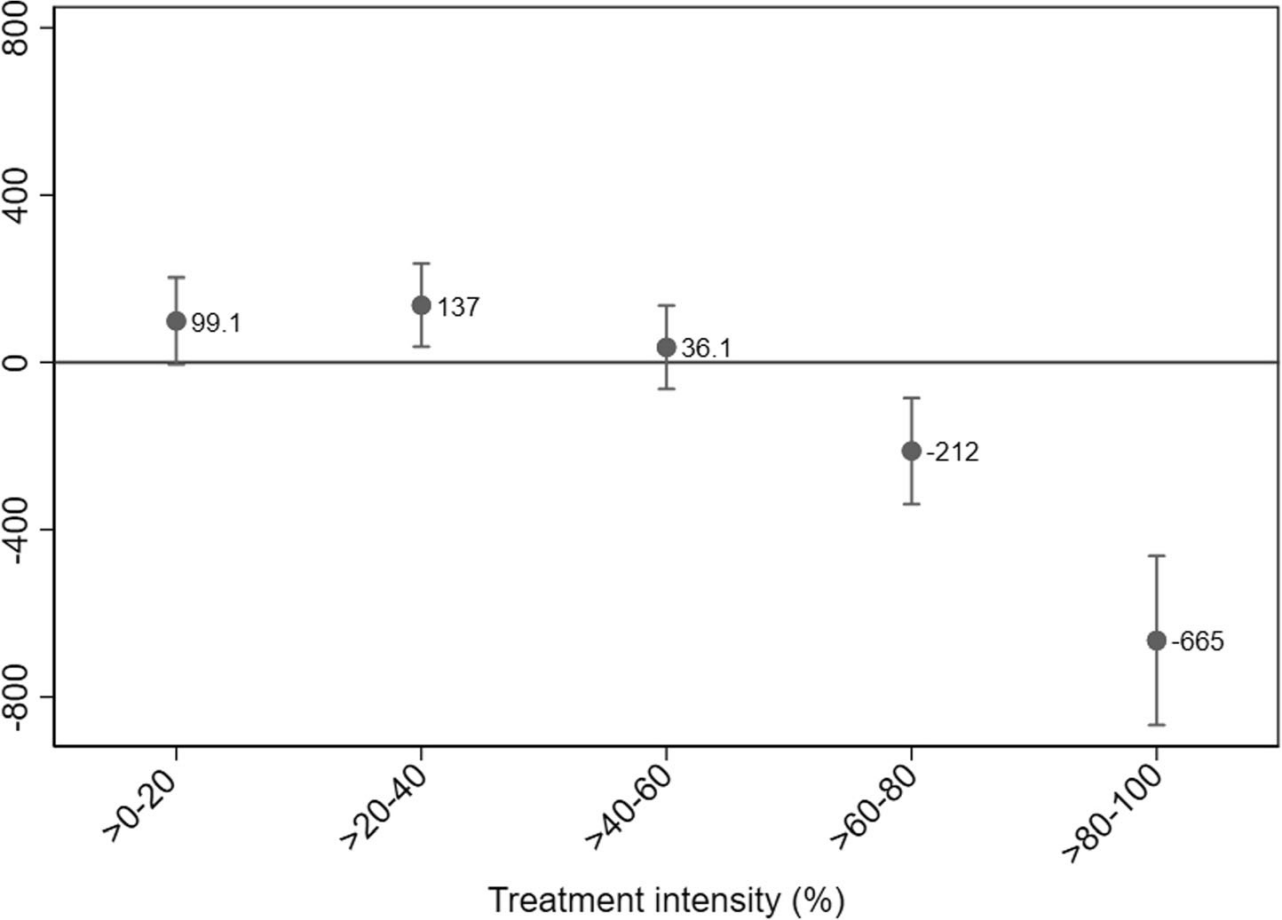
Effect on the total gross wages among incumbent employees with a minimum age of 26 by treatment intensity. DDD estimation. Notes: Dependent variable: Gross wage sum for incumbent employees with a minimum age of 26 (measured in 100SEK). Treatment period: 2006–2008. Underlying time period: 2003–2005. Within-firm estimation. Only surviving firms with at least one employee per year are included. Outliers (defined as extreme annual changes in employment and/or wages) are excluded. Employment outliers are defined as annual employment changes of more than three standard deviations from average change (+/- 88 employees). Wage outliers are defined as annual changes in the gross wage sum of more than three standard deviations from the average change. Firm clustered standard errors. Point estimates with 95% confidence intervals

In Fig. 7, we estimate the effect on the total wage sum for incumbent employees with a minimum age of 26 using our DDD model.^20^ The point estimates suggest an overall inverse relationship between firms’ labor cost savings and the wage development for incumbent employees. Total wages for older (nontargeted) workers in firms within the >20–40 treatment intensity range increased by 13,700 SEK (1480 USD) due to the payroll tax reform, which corresponds to an average wage increase per worker of 2430 SEK (262 USD) over the 2006–2008 period. On the other hand, total wages decreased for incumbent employees in firms within the >60–80 and >80–100 treatment intensity ranges by, on average, 21,200 SEK (2290 USD) and 66,500 SEK (7182 USD), respectively. These decreases correspond to approximately 2200 SEK (238 USD) and 2028 SEK (219 USD) per incumbent employee.

A naïve interpretation of these negative point estimates is that the payroll tax reform is linked to decreased, rather than increased, wages for incumbent employees at firms with large labor cost savings. However, assessing the underlying DiD estimates, we find wage increases in both the underlying period of 2003–2005 and the reform period of 2006–2008, though the increase was larger in the prereform period. Thus, a more sophisticated interpretation is that the payroll tax reform had a negative impact on the growth in—but not the level of—the total gross wage sum for employees who were incumbent at the treated firms.

A potential explanation for the findings for firms within the lower treatment intensity groups is that the labor cost savings were not sufficient to increase employment along the extensive margin (additional employees), resulting instead in an increase along the intensive margin (number of hours worked by incumbents), which could be the result of the scale (output) effect, through which firms increased their production in response to lower marginal costs. In turn, this effect causes an increase in the wage sum relative to the control firms and the underlying time period. Our finding for the >20–40 group, which suggests a positive and statistically significant effect on the wages of older incumbents, is in line with the Saez et al. (2019a) finding of rent sharing, i.e., that the payroll tax reform resulted in some wage increases for nontargeted individuals. By contrast, a possible interpretation of our results for firms with larger labor cost savings is that some work tasks previously performed by older incumbent employees were overtaken by new, younger employees, resulting in a decrease in the number of hours worked by incumbents and, consequently, a lower wage sum for the incumbents due to the reform.^21^

## Summary and Discussion

In the Scandinavian welfare states, minimum wages are set in negotiations between the employers’ organizations and the trade unions. Politicians have very limited opportunities to influence minimum wages within this institutional context. Under such circumstances, payroll taxes represent one opportunity for politicians to influence the labor costs of employers and thus their labor demand. However, numerous studies have argued that payroll tax cuts are inefficient since they result in increased wages rather than increased employment (Holmlund 1983; Gruber 1997). The question whether payroll tax cuts are efficient in increasing employment is of particular interest due to the outbreak of COVID-19, with numerous countries reducing payroll taxes in response to the economic downturn following the pandemic (Gentilini et al., 2020).

We investigated the efficiency of a payroll tax cut in Sweden that lowered payroll taxes for employees aged 19–25 years by 11 percentage points. The reform was designed so that employers received a reduced payroll tax for all individuals aged 19–25 who were employed at the firm when the reform was implemented. Theoretically, the reform gave rise to both a substitution effect and a scale effect. On the one hand, the substitution effect should have incentivized employers to recruit young individuals since they became less costly to hire. On the other hand, the labor cost savings reduced employers’ marginal cost of production and created a scale effect through which employers could have recruited more senior employees who were not explicitly targeted by the reform.

In our empirical analysis we explicitly considered that there was a variation in treatment intensity between the firms based on how many young employees they had at the time of the reform. Employers who had many young employees received a large reduction in labor costs, i.e., a high treatment intensity, while employers who had few young employees generally received a smaller reduction in their labor costs. We used this between-firm variation in treatment intensity to evaluate the effects of the youth payroll tax cut on both the number of employees and the total wages among incumbent workers.

Our empirical analysis is based on matched employer-employee data from Statistics Sweden, which covers all residents in Sweden that are at least 16 years old. To account for the fact that firms with a high treatment intensity are larger and tend to grow more in absolute terms than small firms, we estimated a difference-in-difference-in-differences (DDD) model. Unlike an ordinary difference-in-difference (DiD) model, the DDD model accounts for any factor that could have caused the trends in the outcome variable to differ between the treated and control firms prior to the reform.

Our results showed that most employers received a small reduction in labor costs when the youth payroll tax cut was implemented in 2007. However, employers who received a large reduction in labor costs increased their employment significantly more than employers who received marginal reductions or no reductions at all. This finding is in line with the Saez et al. (2019) findings that the employment effect of the youth payroll tax cut varied with firms’ exposure to the reform. However, in contrast to Saez et al. (2019), we relied on an absolute treatment intensity measure that made it possible to estimate the total number of jobs created by the reform. In total, we found that the 2007 payroll tax reform created 18,100 jobs over the period of 2006-2008. This is a considerably larger job creation than previously found by Egebark and Kaunitz (2013), who analyzed the reform from an individual-level perspective and estimated that 6–10,000 jobs per year were created within the targeted age group.

One concern when implementing payroll tax reforms is that insiders can take advantage of the labor cost reductions by increasing their wages at the expense of outsiders’ possibilities to enter employment. We have therefore also investigated the effect of the youth payroll tax reform on the gross wages for older incumbent workers, finding an inverse relationship between the size of firms’ labor cost savings and the wage development for incumbent workers with a minimum age of 26. One possible interpretation of these findings is that firms with small labor cost savings utilized their savings to increase the number of work hours for incumbent workers, which is in line with the Saez et al. (2019a) finding that the reform benefitted individuals not explicitly targeted by the reform. However, for firms with larger labor cost savings, we found a negative impact on the wages of older incumbents. In combination with an increased number of young employees, this suggested that some work tasks previously performed by the incumbents were overtaken by young employees, causing a reduction in the incumbents’ number of hours worked and, consequently, in their total wages.

As discussed, reduced payroll taxes have been argued to predominantly result in increased wages rather than increased employment (Holmlund 1983; Gruber 1997). Our findings do not support that claim. A potential explanation is that the Swedish wage negotiations take place at the industry level, while the evaluated payroll tax reform generated firm-level labor cost savings which varied extensively both within and across industries. In turn, this could have made it difficult to incorporate the savings into the wage negotiations, thereby limiting the effect on wages.

There are some interesting directions for future research. First, it would be of interest to investigate to what extent new employees entered from a previous period of employment or unemployment or were previously outside the labor force. Second, we believe that more research is needed on how payroll tax reforms influence the labor incomes of those already employed and to what extent it hinders outsiders’ possibility to enter employment.

## Supplementary Information

ESM 1 (DOCX 179 kb)

## Footnotes

1 As of April 15, 2019, 1 SEK corresponds to approximately 0.108 USD.

2 Approximately one third of the payroll tax is unrelated to the financing of social benefits.

3 To be precise, the payroll tax was reduced from 32.420 to 21.315%. Using two decimals, the previous government stated the new payroll tax level to be 21.31, yielding a reduction of 11.11 percentage points (Swedish Government 2006). However, during the second half of 2007, the reduction was limited to 9.71 percentage points, and thus, the reform was not fully implemented until the start of year 2008.

4 An income base amount is, for instance, used to calculate the pension-qualifying income. Over the 2003-2008 period, the income base amount ranges between 40,900 SEK (4,417 USD) and 48,000 SEK (5,184 USD).

5 It should be noted that we observe the gross wages received from each individual’s primary employer (largest income source) throughout the year, while the employment variables are measured in the month of November, which entails that the firm-level gross wages are built upon individuals for whom the employer in November is also the primary employer throughout the year. Thus, we will underestimate the firm-level gross wages if some employees have another primary employer.

6 More specifically, all firms that had an annual employment change corresponding to at least +/- three standard deviations from the average change are excluded. This is equivalent to an annual employment change of +/- 88 employees. Over the 2003–2008 period, 1539 of 744,032 firms are considered outliers.

7 For an individual to be assigned industry code 0, (s)he has to be either hired by a municipality while having an unclear work status (e.g., participation in labor market policies) or receive labor income from a firm not registered in the Statistical Business Register (e.g., a firm operating abroad). In addition, industry code 0 could in some instances reflect missing information on industry affiliation.

8 To be exact, Saez et al. (2019a) use the 2006 gross wage share for 19–25-year-olds (the eligible age group in 2007).

9 To test this, we plot the distributions of the labor cost savings in 2006 and 2007. We find the distributions to be very similar, suggesting that the 2006 distribution closely resembles the actual 2007 savings. However, the 2007 distribution is slightly skewed to the right, implying that the employment of, and total wages for, youth have increased. As such, there is reason to believe that using the cost savings of 2007 would give rise to selection bias as some firms self-select into treatment. See Figure A2 in Appendix.

10 The reason for limiting our analysis to the time period 2006–2008 is twofold. First, analyzing the employment effects of the further reform extension in 2009 would be difficult since firms could self-select into treatment once the reform was implemented. Second, the likelihood of noise affecting our results increases with the length of the post-treatment period (Mian and Sufi 2012).

11 For the 2003–2005 DiD-estimation, we calculate (placebo) treatment intensities using data for 2003. The differences in the average firm size between the treatment and control groups are convincingly similar in 2003 and 2006 (compare Table A1 and Table 2).

12 See Figure A1 in the Appendix and the discussion in Section 5.1 below.

13 Technically, 2004–2005 and 2007–2008 are jointly defined as the post-treatment periods of the time periods 2003–2005 and 2006–2008, respectively. Thus, the DDD model does not capture the change from 2006 to 2008 but instead the average change over 2006–2008, where 2007–2008 is considered a joint post-treatment period, which means that the changes from 2006–2007 (and 2003–2004) are also taken into consideration in the DDD estimation.

14 See, e.g., Gravelle and Rees (2004) for the theory and derivations related to substitution and output effects in the context of price changes of firms’ input factors.

15 Egebark and Kaunitz (2013) also refer to this as a scale effect. They acknowledge that if comparing individuals within different age groups, the magnitude of this scale effect increases with an individual’s age, assuming that firms prefer to hire more experienced (older) employees. Saez et al. (2019a) refer to the downward shift in marginal costs as the cost channel and, moreover, emphasize that it is more prominent among youth-intensive firms.

16 One example would be a firm that hires some eligible, young workers but additionally decides to use part of the labor cost savings to recruit someone more experienced to tutor and supervise the less experienced workers.

17 We also estimated model (1) using firm-level random effects instead of fixed effects. Treating unobserved firm-level heterogeneity as random effects instead of fixed effects has a negligible impact on the estimates. See the regression tables in the Appendix.

18 The corresponding regression tables for our DDD estimations are presented in Table A2 in the Appendix. The estimates in the figure are represented by the average treatment effect (ATE) estimate in the fifth column of each table.

19 This number is derived by relating the point estimates to the corresponding number of firms, i.e., 0.127*10,878+0.141*10,864+0.119*10,854+0.38*10,878+0.902*10,866=18,140 jobs.

20 Complete regression tables are presented in Appendix (see table A5)

21 We lack data on the number of working hours and therefore cannot examine the intensive margin effects in detail.
