# Supplementary material for: How Do Firms Respond to Reduced Labor Costs? Evidence from the 2007 Swedish Payroll Tax Reform
Source: J Ind Compet Trade. 2021 Mar 4;21(3):315–38. doi: 10.1007/s10842-021-00356-6 (PMC7931164; doi:10.1007/s10842-021-00356-6)
Supplement: Supplementary file 1 — (DOCX 179 kb) [file 10842_2021_356_MOESM1_ESM.docx]

**APPENDIX**

**Figure A1.** Employment effects by treatment intensity. DiD estimation.

**
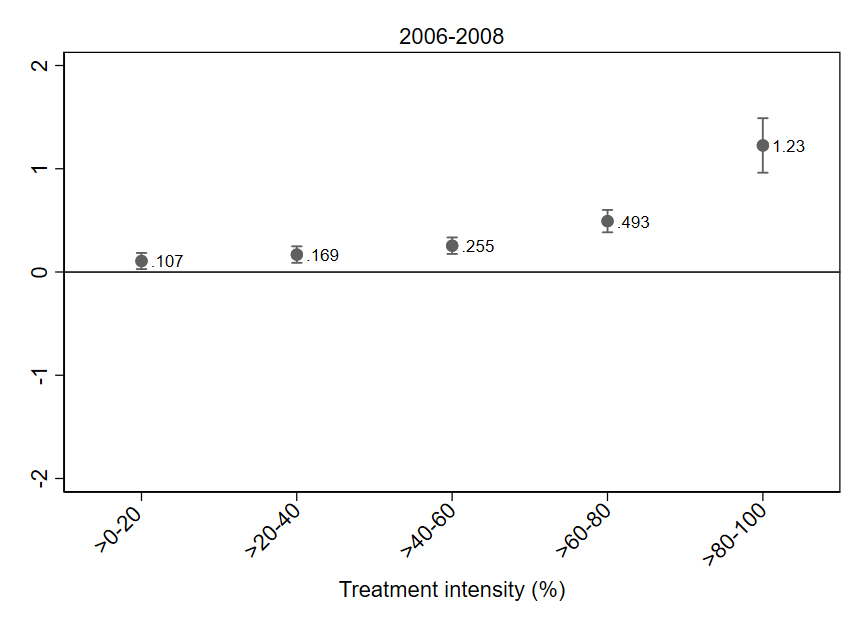
**

**
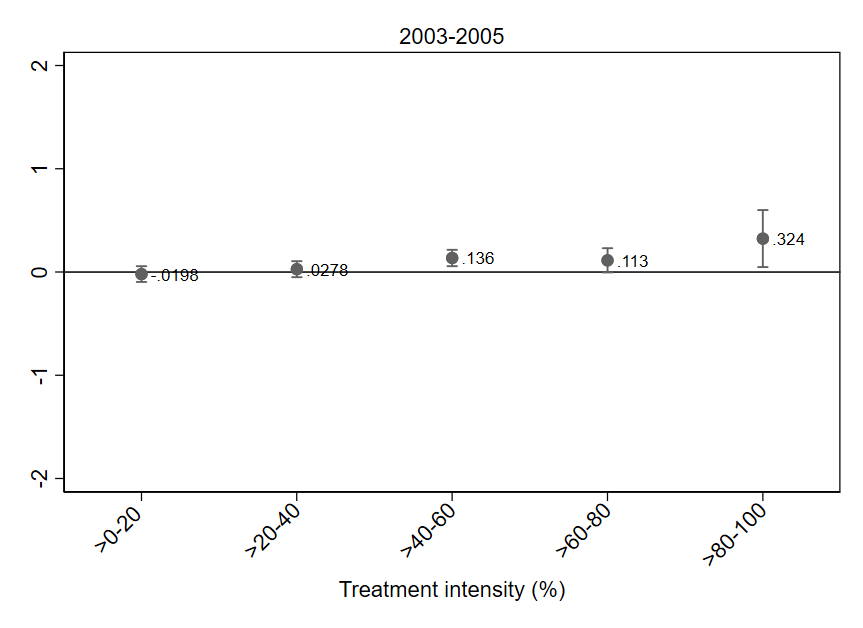
**

Notes: Dependent variable: Firm size (no. employees). Treatment period: 2006-2008. Underlying time period: 2003-2005. Within-firm estimation. Only surviving firms with at least one employee per year are included. Outliers (defined as annual employment changes of more than three standard deviations from the average change (+/- 88 employees)) are excluded. Firm clustered standard errors. Point estimates with 95 % confidence intervals.

**Figure A2.** Labor cost savings in 2006 and 2007.

**
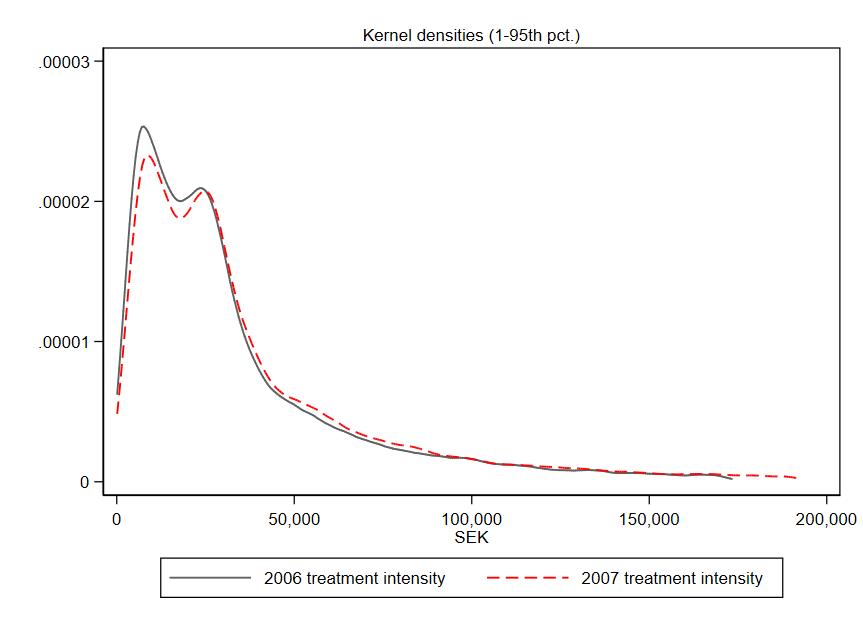
**

Notes: This figure shows the firm-level distribution of the estimated and actual labor cost savings in 2006 and 2007, respectively. The savings are measured according to the price level of 2016. Including the 1-95^th^ percentile of each distribution.

**Figure A3.** Employment effects among firms within the >80-100 treatment intensity range. Split into four groups. DDD estimation.


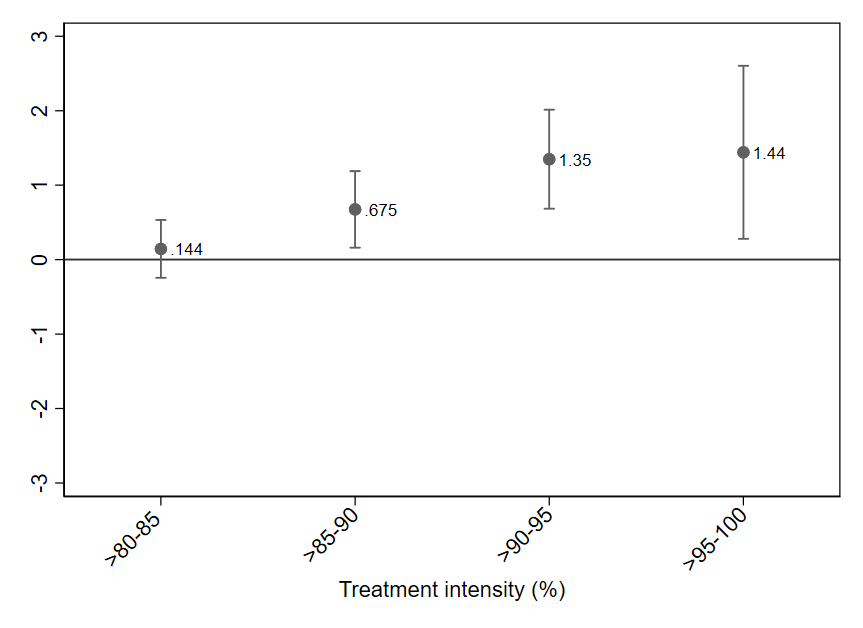


Notes. Dependent variable: Firm size (number of employees). Treatment period: 2006-2008. Underlying time period: 2003-2005. Within-firm estimation. Only surviving firms with at least one employee per year are included. Outliers (defined as annual employment changes of more than three standard deviations from average change (+/- 88 employees)) are excluded. Firm clustered standard errors. Point estimates with 95 % confidence intervals.

**Table A1.** Firm size in 2003 by treatment and control groups for the time period 2003-2005.

|  | **Mean** | **Median** | **Std.dev.** | **Min** | **Max** | **# Firms** |
| --- | --- | --- | --- | --- | --- | --- |
| **Firm size (# employees)** |  |  |  |  |  |  |
| Control | 2.630 | 1 | 5.466 | 1 | 534 | 207,201 |
| Dose >0-20 % | 8.248 | 4 | 18.069 | 1 | 1,101 | 9,696 |
| Dose >20-40 % | 8.765 | 5 | 15.799 | 1 | 353 | 9,733 |
| Dose >40-60 % | 10.885 | 6 | 18.450 | 1 | 545 | 9,719 |
| Dose >60-80 % | 17.741 | 10 | 29.902 | 1 | 812 | 9,725 |
| Dose >80-100 % | 79.526 | 31 | 172.981 | 1 | 2,754 | 9,717 |
| **# 19-25 yrs old** |  |  |  |  |  |  |
| Control | 0.031 | 0 | 0.189 | 0 | 6 | 207,201 |
| Dose >0-20 % | 1.160 | 1 | 0.860 | 0 | 13 | 9,696 |
| Dose >20-40 % | 1.474 | 1 | 1.039 | 0 | 24 | 9,733 |
| Dose >40-60 % | 1.598 | 1 | 1.077 | 0 | 18 | 9,719 |
| Dose >60-80 % | 2.714 | 2 | 1.703 | 0 | 28 | 9,725 |
| Dose >80-100 % | 11.530 | 7 | 17.727 | 1 | 410 | 9,717 |
| **# >25 yrs old** |  |  |  |  |  |  |
| Control | 2.595 | 1 | 5.415 | 0 | 532 | 207,201 |
| Dose >0-20 % | 6.846 | 3 | 17.873 | 0 | 1,098 | 9,696 |
| Dose >20-40 % | 7.147 | 3 | 15.389 | 0 | 352 | 9,733 |
| Dose >40-60 % | 9.160 | 4 | 18.082 | 0 | 539 | 9,719 |
| Dose >60-80 % | 14.766 | 7 | 29.405 | 0 | 808 | 9,725 |
| Dose >80-100 % | 66.900 | 22 | 160.066 | 0 | 2,497 | 9,717 |

Note: Includes surviving firms that have at least one employee per year over 2003-2005. *Outliers (defined as annual employment changes of more than three standard deviations from average change (+/-88 employees) are excluded*.

**Table A2.** 2006-2008 employment effect by treatment intensity. DDD regressions.

| **Specification:** | **1** | **2** | **3** | **4** | **5** | **6** | **Specification:** | **1** | **2** | **3** | **4** | **5** | **6** |
| --- | --- | --- | --- | --- | --- | --- | --- | --- | --- | --- | --- | --- | --- |
| **Tr. intensity:** | *0-20* | *0-20* | *0-20* | *0-20* | *0-20* | *0-20* | **Tr. intensity:** | *20-40* | *20-40* | *20-40* | *20-40* | *20-40* | *20-40* |
| Time | 0.162*** | 0.162*** | 0.159*** | 0.161*** | 0.162*** | 0.162*** | Time | 0.162*** | 0.162*** | 0.159*** | 0.162*** | 0.162*** | 0.162*** |
|  | (0.0189) | (0.0142) | (0.00390) | (0.00391) | (0.00369) | (0.00369) |  | (0.0184) | (0.0142) | (0.00391) | (0.00393) | (0.00369) | (0.00369) |
| Group | 5.617*** | 5.617*** | 5.126*** | 5.104*** | 0.628*** | 0.933*** | Group | 6.134*** | 6.134*** | 5.622*** | 5.595*** | 0.554*** | 0.936*** |
|  | (0.0730) | (0.518) | (0.176) | (0.175) | (0.0745) | (0.0719) |  | (0.0709) | (0.439) | (0.145) | (0.145) | (0.0663) | (0.0641) |
| Treat | -0.0857*** | -0.0857*** | -0.0621*** | -0.0619*** | 0.0641*** | 0.0600*** | Treat | -0.0857*** | -0.0857*** | -0.0654*** | -0.0648*** | 0.0644*** | 0.0602*** |
|  | (0.0215) | (0.0239) | (0.0136) | (0.0136) | (0.00562) | (0.00556) |  | (0.0209) | (0.0239) | (0.0136) | (0.0136) | (0.00562) | (0.00556) |
| Group*Time | -0.0198 | -0.0198 | -0.0320 | -0.0335 | -0.0198 | -0.0198 | Group*Time | 0.0278 | 0.0278 | 0.00587 | 0.00198 | 0.0278 | 0.0278 |
|  | (0.0894) | (0.0518) | (0.0393) | (0.0393) | (0.0391) | (0.0391) |  | (0.0868) | (0.0651) | (0.0404) | (0.0404) | (0.0395) | (0.0395) |
| Time*Treat | 0.0333 | 0.0333** | 0.0320*** | 0.0309*** | 0.0333*** | 0.0333*** | Time*Treat | 0.0333 | 0.0333** | 0.0323*** | 0.0310*** | 0.0333*** | 0.0333*** |
|  | (0.0263) | (0.0132) | (0.00545) | (0.00546) | (0.00516) | (0.00516) |  | (0.0256) | (0.0132) | (0.00546) | (0.00547) | (0.00516) | (0.00516) |
| Group*Treat | 0.383*** | 0.383 | 0.314 | 0.296 | 0.349*** | 0.327*** | Group*Treat | 0.386*** | 0.386 | 0.363* | 0.350* | 0.579*** | 0.533*** |
|  | (0.100) | (0.283) | (0.235) | (0.235) | (0.0888) | (0.0866) |  | (0.0976) | (0.249) | (0.199) | (0.198) | (0.0835) | (0.0813) |
| ATE | 0.127 | 0.127 | 0.131** | 0.128** | 0.127** | 0.127** | ATE | 0.141 | 0.141 | 0.165*** | 0.169*** | 0.141** | 0.141** |
|  | (0.123) | (0.103) | (0.0566) | (0.0566) | (0.0557) | (0.0557) |  | (0.120) | (0.0923) | (0.0575) | (0.0575) | (0.0567) | (0.0567) |
| Constant | 2.630*** | 2.630*** | 1.097*** | 1.692*** | 2.782*** | 2.862*** | Constant | 2.630*** | 2.630*** | 1.080*** | 1.623*** | 2.804*** | 2.888*** |
|  | (0.0154) | (0.108) | (0.0174) | (0.200) | (0.00465) | (0.0138) |  | (0.0150) | (0.108) | (0.0186) | (0.190) | (0.00455) | (0.0133) |
| Observations | 1,349,253 | 1,349,253 | 1,349,253 | 1,349,253 | 1,349,253 | 1,349,253 | Observations | 1,349,322 | 1,349,322 | 1,349,322 | 1,349,322 | 1,349,322 | 1,349,322 |
| R-squared | 0.030 | 0.030 | 0.135 | 0.140 | 0.008 | . | R-squared | 0.037 | 0.037 | 0.160 | 0.165 | 0.010 | . |
| Firm clustered s.e. | No | No | Yes | Yes | Yes | Yes | Firm clustered s.e | No | No | Yes | Yes | Yes | Yes |
| Industry clustered s.e. | No | Yes | No | No | No | No | Industry clustered s.e | No | Yes | No | No | No | No |
| Firm FE | No | No | No | No | Yes | No | Firm FE | No | No | No | No | Yes | No |
| Firm RE | No | No | No | No | No | Yes | Firm RE | No | No | No | No | No | Yes |
| Industry FE | No | No | Yes | Yes | No | No | Industry FE | No | No | Yes | Yes | No | No |
| Municip FE | No | No | No | Yes | No | No | Municip FE | No | No | No | Yes | No | No |

Notes. Dependent variable: Firm size (no. employees). Treatment period: 2006-2008. Underlying time period: 2003-2005. Only surviving firms with at least one employee per year are included. The point estimates in the figures are represented by the ATE estimate in the fifth column. DDD estimation.*p<0.1, **p<0.05, ***p<0.01

**Cont. - Table A2.** 2006-2008 employment effect by treatment intensity. DDD regressions.

| **Specification:** | **1** | **2** | **3** | **4** | **5** | **6** | **Specification:** | **1** | **2** | **3** | **4** | **5** | **6** |
| --- | --- | --- | --- | --- | --- | --- | --- | --- | --- | --- | --- | --- | --- |
| **Tr. intensity:** | *40-60* | *40-60* | *40-60* | *40-60* | *40-60* | *40-60* | **Tr. intensity:** | *60-80* | *60-80* | *60-80* | *60-80* | *60-80* | *60-80* |
| Time | 0.162*** | 0.162*** | 0.159*** | 0.162*** | 0.162*** | 0.162*** | Time | 0.162*** | 0.162*** | 0.157*** | 0.160*** | 0.162*** | 0.162*** |
|  | (0.0194) | (0.0142) | (0.00387) | (0.00389) | (0.00369) | (0.00369) |  | (0.0230) | (0.0142) | (0.00401) | (0.00403) | (0.00369) | (0.00369) |
| Group | 8.255*** | 8.255*** | 7.467*** | 7.438*** | 0.475*** | 0.961*** | Group | 15.11*** | 15.11*** | 14.13*** | 14.06*** | 1.184*** | 2.275*** |
|  | (0.0750) | (0.493) | (0.169) | (0.169) | (0.0789) | (0.0764) |  | (0.0887) | (0.887) | (0.281) | (0.281) | (0.137) | (0.132) |
| Treat | -0.0857*** | -0.0857*** | -0.0664*** | -0.0648*** | 0.0654*** | 0.0613*** | Treat | -0.0857*** | -0.0857*** | -0.0727*** | -0.0721*** | 0.0658*** | 0.0621*** |
|  | (0.0221) | (0.0239) | (0.0136) | (0.0137) | (0.00566) | (0.00560) |  | (0.0261) | (0.0239) | (0.0138) | (0.0138) | (0.00570) | (0.00565) |
| Group*Time | 0.136 | 0.136** | 0.129*** | 0.126*** | 0.136*** | 0.136*** | Group*Time | 0.113 | 0.113 | 0.0860 | 0.0835 | 0.113* | 0.113* |
|  | (0.0918) | (0.0632) | (0.0408) | (0.0408) | (0.0405) | (0.0405) |  | (0.109) | (0.130) | (0.0608) | (0.0606) | (0.0600) | (0.0600) |
| Time*Treat | 0.0333 | 0.0333** | 0.0335*** | 0.0323*** | 0.0333*** | 0.0333*** | Time*Treat | 0.0333 | 0.0333** | 0.0347*** | 0.0333*** | 0.0333*** | 0.0333*** |
|  | (0.0270) | (0.0132) | (0.00545) | (0.00546) | (0.00516) | (0.00516) |  | (0.0320) | (0.0132) | (0.00562) | (0.00564) | (0.00516) | (0.00516) |
| Group*Treat | 0.151 | 0.151 | 0.297 | 0.300 | 0.810*** | 0.792*** | Group*Treat | -0.853*** | -0.853** | -0.731** | -0.725** | 1.276*** | 1.226*** |
|  | (0.103) | (0.301) | (0.238) | (0.238) | (0.0964) | (0.0945) |  | (0.122) | (0.411) | (0.354) | (0.354) | (0.135) | (0.133) |
| ATE | 0.119 | 0.119 | 0.122** | 0.125** | 0.119** | 0.119** | ATE | 0.380** | 0.380** | 0.407*** | 0.409*** | 0.380*** | 0.380*** |
|  | (0.126) | (0.0942) | (0.0582) | (0.0582) | (0.0576) | (0.0576) |  | (0.150) | (0.172) | (0.0825) | (0.0823) | (0.0808) | (0.0808) |
| Constant | 2.630*** | 2.630*** | 1.042*** | 1.673*** | 2.892*** | 2.978*** | Constant | 2.630*** | 2.630*** | 1.049*** | 1.551*** | 3.138*** | 3.235*** |
|  | (0.0159) | (0.108) | (0.0198) | (0.235) | (0.00487) | (0.0141) |  | (0.0188) | (0.108) | (0.0220) | (0.199) | (0.00627) | (0.0172) |
| Observations | 1,349,250 | 1,349,250 | 1,349,250 | 1,349,250 | 1,349,250 | 1,349,250 | Observations | 1,349,340 | 1,349,340 | 1,349,340 | 1,349,340 | 1,349,340 | 1,349,340 |
| R-squared | 0.057 | 0.057 | 0.171 | 0.175 | 0.010 | . | R-squared | 0.117 | 0.117 | 0.216 | 0.220 | 0.017 | . |
| Firm clustered s.e | No | No | Yes | Yes | Yes | Yes | Firm clustered s.e | No | No | Yes | Yes | Yes | Yes |
| Industry clustered s.e | No | Yes | No | No | No | No | Industry clustered s.e | No | Yes | No | No | No | No |
| Firm FE | No | No | No | No | Yes | No | Firm FE | No | No | No | No | Yes | No |
| Firm RE | No | No | No | No | No | Yes | Firm RE | No | No | No | No | No | Yes |
| Industry FE | No | No | Yes | Yes | No | No | Industry FE | No | No | Yes | Yes | No | No |
| Municip FE | No | No | No | Yes | No | No | Municip FE | No | No | No | Yes | No | No |

Notes. Dependent variable: Firm size (no. employees). Treatment period: 2006-2008. Underlying time period: 2003-2005. Only surviving firms with at least one employee per year are included. The point estimates in the figures are represented by the ATE estimate in the fifth column. DDD estimation. *p<0.1, **p<0.05, ***p<0.01

**Cont. - Table A2.** 2006-2008 employment effect by treatment intensity. DDD regressions.

| **Specification:** | **1** | **2** | **3** | **4** | **5** | **6** |
| --- | --- | --- | --- | --- | --- | --- |
| **Tr. intensity:** | *80-100* | *80-100* | *80-100* | *80-100* | *80-100* | *80-100* |
| Time | 0.162* | 0.162*** | 0.149*** | 0.150*** | 0.162*** | 0.162*** |
|  | (0.0980) | (0.0142) | (0.00748) | (0.00757) | (0.00369) | (0.00369) |
| Group | 76.90*** | 76.90*** | 64.10*** | 63.98*** | 5.457*** | 9.434*** |
|  | (0.378) | (11.20) | (1.182) | (1.183) | (0.533) | (0.520) |
| Treat | -0.0857 | -0.0857*** | -0.177*** | -0.166*** | 0.0565*** | 0.0533*** |
|  | (0.111) | (0.0239) | (0.0290) | (0.0292) | (0.00591) | (0.00588) |
| Group*Time | 0.324 | 0.324 | 0.0925 | 0.0939 | 0.324** | 0.324** |
|  | (0.463) | (0.939) | (0.220) | (0.220) | (0.141) | (0.141) |
| Time*Treat | 0.0333 | 0.0333** | 0.0378*** | 0.0372*** | 0.0333*** | 0.0333*** |
|  | (0.136) | (0.0132) | (0.0108) | (0.0109) | (0.00516) | (0.00516) |
| Group*Treat | -5.689*** | -5.689*** | -3.324*** | -3.298*** | 4.139*** | 4.088*** |
|  | (0.520) | (1.803) | (0.721) | (0.721) | (0.317) | (0.316) |
| ATE | 0.902 | 0.902 | 1.320*** | 1.331*** | 0.902*** | 0.902*** |
|  | (0.637) | (1.405) | (0.310) | (0.310) | (0.187) | (0.187) |
| Constant | 2.630*** | 2.630*** | 0.983*** | 1.023 | 5.589*** | 4.969*** |
|  | (0.0800) | (0.108) | (0.0619) | (0.646) | (0.0217) | (0.0600) |
| Observations | 1,349,280 | 1,349,280 | 1,349,280 | 1,349,280 | 1,349,280 | 1,349,280 |
| R-squared | 0.154 | 0.154 | 0.353 | 0.354 | 0.031 | . |
| Firm clustered s.e | No | No | Yes | Yes | Yes | Yes |
| Industry clustered s.e | No | Yes | No | No | No | No |
| Firm FE | No | No | No | No | Yes | No |
| Firm RE | No | No | No | No | No | Yes |
| Industry FE | No | No | Yes | Yes | No | No |
| Municip FE | No | No | No | Yes | No | No |

Notes. Dependent variable: Firm size (no. employees). Treatment period: 2006-2008. Underlying time period: 2003-2005. Only surviving firms with at least one employee per year are included. The point estimates in the figures are represented by the ATE estimate in the fifth column. DDD estimation. *p<0.1, **p<0.05, ***p<0.01

**Table A3.** 2006-2008 employment effect for 19-25-year-olds by treatment intensity. DDD regressions.

| **Specification:** | **1** | **2** | **3** | **4** | **5** | **6** | **Specification:** | **1** | **2** | **3** | **4** | **5** | **6** |
| --- | --- | --- | --- | --- | --- | --- | --- | --- | --- | --- | --- | --- | --- |
| **Tr. intensity:** | *0-20* | *0-20* | *0-20* | *0-20* | *0-20* | *0-20* | **Tr. intensity:** | *20-40* | *20-40* | *20-40* | *20-40* | *20-40* | *20-40* |
| Time | 0.0661*** | 0.0661*** | 0.0660*** | 0.0661*** | 0.0661*** | 0.0661*** | Time | 0.0661*** | 0.0661*** | 0.0659*** | 0.0660*** | 0.0661*** | 0.0661*** |
|  | (0.00132) | (0.00522) | (0.000857) | (0.000858) | (0.000860) | (0.000860) |  | (0.00138) | (0.00522) | (0.000857) | (0.000857) | (0.000860) | (0.000860) |
| Group | 1.129*** | 1.129*** | 1.085*** | 1.084*** | 0.590*** | 0.933*** | Group | 1.443*** | 1.443*** | 1.395*** | 1.393*** | 0.783*** | 1.199*** |
|  | (0.00509) | (0.0219) | (0.00860) | (0.00859) | (0.0132) | (0.00872) |  | (0.00532) | (0.0304) | (0.0101) | (0.0101) | (0.0134) | (0.00975) |
| Treat | -0.000278 | -0.000278 | 0.000529 | 0.000609 | 0.0140*** | 0.00830*** | Treat | -0.000278 | -0.000278 | 0.000403 | 0.000501 | 0.0139*** | 0.00847*** |
|  | (0.00150) | (0.000740) | (0.000588) | (0.000590) | (0.000843) | (0.000665) |  | (0.00157) | (0.000740) | (0.000594) | (0.000596) | (0.000846) | (0.000676) |
| Group*Time | -0.177*** | -0.177*** | -0.177*** | -0.177*** | -0.177*** | -0.177*** | Group*Time | -0.211*** | -0.211*** | -0.212*** | -0.212*** | -0.211*** | -0.211*** |
|  | (0.00624) | (0.0175) | (0.0131) | (0.0131) | (0.0131) | (0.0131) |  | (0.00651) | (0.0150) | (0.0132) | (0.0132) | (0.0132) | (0.0132) |
| Time*Treat | 0.0152*** | 0.0152*** | 0.0152*** | 0.0152*** | 0.0152*** | 0.0152*** | Time*Treat | 0.0152*** | 0.0152*** | 0.0151*** | 0.0151*** | 0.0152*** | 0.0152*** |
|  | (0.00183) | (0.00200) | (0.00124) | (0.00124) | (0.00124) | (0.00124) |  | (0.00192) | (0.00200) | (0.00124) | (0.00124) | (0.00124) | (0.00124) |
| Group*Treat | 0.0370*** | 0.0370** | 0.0356*** | 0.0347*** | 0.103*** | 0.0538*** | Group*Treat | 0.0392*** | 0.0392** | 0.0388*** | 0.0384*** | 0.0667*** | 0.0272** |
|  | (0.00701) | (0.0183) | (0.0128) | (0.0128) | (0.0175) | (0.0122) |  | (0.00732) | (0.0153) | (0.0142) | (0.0142) | (0.0185) | (0.0132) |
| ATE | 0.0861*** | 0.0861*** | 0.0857*** | 0.0856*** | 0.0861*** | 0.0861*** | ATE | 0.0873*** | 0.0873*** | 0.0881*** | 0.0883*** | 0.0873*** | 0.0873*** |
|  | (0.00858) | (0.0233) | (0.0188) | (0.0188) | (0.0188) | (0.0188) |  | (0.00897) | (0.0244) | (0.0199) | (0.0199) | (0.0199) | (0.0199) |
| Constant | 0.0306*** | 0.0306*** | -0.0306*** | -0.0262*** | 0.0463*** | 0.0469*** | Constant | 0.0306*** | 0.0306*** | -0.0317*** | -0.0262*** | 0.0528*** | 0.0502*** |
|  | (0.00108) | (0.00154) | (0.00213) | (0.00918) | (0.000747) | (0.000571) |  | (0.00113) | (0.00154) | (0.00214) | (0.00870) | (0.000753) | (0.000596) |
| Observations | 1,349,253 | 1,349,253 | 1,349,253 | 1,349,253 | 1,349,253 | 1,349,253 | Observations | 1,349,322 | 1,349,322 | 1,349,322 | 1,349,322 | 1,349,322 | 1,349,322 |
| R-squared | 0.174 | 0.174 | 0.189 | 0.191 | 0.036 | . | R-squared | 0.237 | 0.237 | 0.255 | 0.256 | 0.046 | . |
| Firm clustered s.e | No | No | Yes | Yes | Yes | Yes | Firm clustered s.e | No | No | Yes | Yes | Yes | Yes |
| Industry clustered s.e | No | Yes | No | No | No | No | Industry clustered s.e | No | Yes | No | No | No | No |
| Firm FE | No | No | No | No | Yes | No | Firm FE | No | No | No | No | Yes | No |
| Firm RE | No | No | No | No | No | Yes | Firm RE | No | No | No | No | No | Yes |
| Industry FE | No | No | Yes | Yes | No | No | Industry FE | No | No | Yes | Yes | No | No |
| Municip FE | No | No | No | Yes | No | No | Municip FE | No | No | No | Yes | No | No |

Notes. Dependent variable: Number of 19-25 year-olds. Treatment period: 2006-2008. Underlying time period: 2003-2005. Only surviving firms with at least one employee per year are included. The point estimates in the figures are represented by the ATE estimate in the fifth column. DDD estimation.

*p<0.1, **p<0.05, ***p<0.01

**Cont. - Table A3.** 2006-2008 employment effect for 19-25-year-olds by treatment intensity. DDD regressions.

| **Specification:** | **1** | **2** | **3** | **4** | **5** | **6** | **Specification:** | **1** | **2** | **3** | **4** | **5** | **6** |
| --- | --- | --- | --- | --- | --- | --- | --- | --- | --- | --- | --- | --- | --- |
| **Tr. intensity:** | *40-60* | *40-60* | *40-60* | *40-60* | *40-60* | *40-60* | **Tr. intensity:** | *60-80* | *60-80* | *60-80* | *60-80* | *60-80* | *60-80* |
| Time | 0.0661*** | 0.0661*** | 0.0660*** | 0.0661*** | 0.0661*** | 0.0661*** | Time | 0.0661*** | 0.0661*** | 0.0659*** | 0.0660*** | 0.0661*** | 0.0661*** |
|  | (0.00137) | (0.00522) | (0.000857) | (0.000857) | (0.000860) | (0.000860) |  | (0.00169) | (0.00522) | (0.000858) | (0.000858) | (0.000860) | (0.000860) |
| Group | 1.567*** | 1.567*** | 1.523*** | 1.521*** | 0.810*** | 1.297*** | Group | 2.683*** | 2.683*** | 2.615*** | 2.611*** | 1.224*** | 2.206*** |
|  | (0.00528) | (0.0496) | (0.0106) | (0.0106) | (0.0139) | (0.0105) |  | (0.00653) | (0.106) | (0.0168) | (0.0168) | (0.0238) | (0.0175) |
| Treat | -0.000278 | -0.000278 | 0.000392 | 0.000538 | 0.0143*** | 0.00862*** | Treat | -0.000278 | -0.000278 | 0.000315 | 0.000410 | 0.0140*** | 0.00943*** |
|  | (0.00155) | (0.000740) | (0.000594) | (0.000597) | (0.000853) | (0.000673) |  | (0.00192) | (0.000740) | (0.000616) | (0.000619) | (0.000863) | (0.000705) |
| Group*Time | -0.130*** | -0.130*** | -0.130*** | -0.130*** | -0.130*** | -0.130*** | Group*Time | -0.277*** | -0.277*** | -0.278*** | -0.278*** | -0.277*** | -0.277*** |
|  | (0.00646) | (0.0179) | (0.0126) | (0.0126) | (0.0126) | (0.0126) |  | (0.00799) | (0.0266) | (0.0186) | (0.0186) | (0.0186) | (0.0186) |
| Time*Treat | 0.0152*** | 0.0152*** | 0.0152*** | 0.0152*** | 0.0152*** | 0.0152*** | Time*Treat | 0.0152*** | 0.0152*** | 0.0153*** | 0.0153*** | 0.0152*** | 0.0152*** |
|  | (0.00190) | (0.00200) | (0.00124) | (0.00124) | (0.00124) | (0.00124) |  | (0.00235) | (0.00200) | (0.00124) | (0.00124) | (0.00124) | (0.00124) |
| Group*Treat | 0.0891*** | 0.0891*** | 0.0893*** | 0.0894*** | 0.0138 | 0.0431*** | Group*Treat | 0.0847*** | 0.0847*** | 0.0851*** | 0.0848*** | 0.122*** | 0.102*** |
|  | (0.00727) | (0.0184) | (0.0153) | (0.0152) | (0.0196) | (0.0147) |  | (0.00898) | (0.0253) | (0.0229) | (0.0228) | (0.0295) | (0.0227) |
| ATE | 0.0711*** | 0.0711*** | 0.0707*** | 0.0708*** | 0.0711*** | 0.0711*** | ATE | 0.228*** | 0.228*** | 0.229*** | 0.229*** | 0.228*** | 0.228*** |
|  | (0.00890) | (0.0255) | (0.0192) | (0.0192) | (0.0192) | (0.0192) |  | (0.0110) | (0.0312) | (0.0273) | (0.0273) | (0.0273) | (0.0273) |
| Constant | 0.0306*** | 0.0306*** | -0.0340*** | -0.0302*** | 0.0596*** | 0.0517*** | Constant | 0.0306*** | 0.0306*** | -0.0358*** | -0.0349*** | 0.0892*** | 0.0612*** |
|  | (0.00112) | (0.00154) | (0.00222) | (0.00833) | (0.000784) | (0.000590) |  | (0.00138) | (0.00154) | (0.00258) | (0.0108) | (0.00105) | (0.000714) |
| Observations | 1,349,250 | 1,349,250 | 1,349,250 | 1,349,250 | 1,349,250 | 1,349,250 | Observations | 1,349,340 | 1,349,340 | 1,349,340 | 1,349,340 | 1,349,340 | 1,349,340 |
| R-squared | 0.292 | 0.292 | 0.308 | 0.309 | 0.047 | . | R-squared | 0.434 | 0.434 | 0.447 | 0.448 | 0.052 | . |
| Firm clustered s.e | No | No | Yes | Yes | Yes | Yes | Firm clustered s.e | No | No | Yes | Yes | Yes | Yes |
| Industry clustered s.e | No | Yes | No | No | No | No | Industry clustered s.e | No | Yes | No | No | No | No |
| Firm FE | No | No | No | No | Yes | No | Firm FE | No | No | No | No | Yes | No |
| Firm RE | No | No | No | No | No | Yes | Firm RE | No | No | No | No | No | Yes |
| Industry FE | No | No | Yes | Yes | No | No | Industry FE | No | No | Yes | Yes | No | No |
| Municip FE | No | No | No | Yes | No | No | Municip FE | No | No | No | Yes | No | No |

Notes. Dependent variable: Number of 19-25-year-olds. Treatment period: 2006-2008. Underlying time period: 2003-2005. Only surviving firms with at least one employee per year are included. The point estimates in the figures are represented by the ATE estimate in the fifth column. DDD estimation.

*p<0.1, **p<0.05, ***p<0.01

**Cont. - Table A3.** 2006-2008 employment effect for 19-25-year-olds by treatment intensity. DDD regressions.

| **Specification:** | **1** | **2** | **3** | **4** | **5** | **6** |
| --- | --- | --- | --- | --- | --- | --- |
| **Tr. intensity:** | *80-100* | *80-100* | *80-100* | *80-100* | *80-100* | *80-100* |
| Time | 0.0661*** | 0.0661*** | 0.0654*** | 0.0655*** | 0.0661*** | 0.0661*** |
|  | (0.0105) | (0.00522) | (0.000977) | (0.000984) | (0.000860) | (0.000860) |
| Group | 11.50*** | 11.50*** | 10.42*** | 10.40*** | 2.579*** | 5.670*** |
|  | (0.0406) | (0.829) | (0.154) | (0.153) | (0.116) | (0.108) |
| Treat | -0.000278 | -0.000278 | -0.0100*** | -0.00948*** | 0.0116*** | 0.00924*** |
|  | (0.0120) | (0.000740) | (0.00214) | (0.00219) | (0.000950) | (0.000940) |
| Group*Time | -0.623*** | -0.623*** | -0.652*** | -0.652*** | -0.623*** | -0.623*** |
|  | (0.0497) | (0.0963) | (0.0521) | (0.0521) | (0.0508) | (0.0508) |
| Time*Treat | 0.0152 | 0.0152*** | 0.0147*** | 0.0146*** | 0.0152*** | 0.0152*** |
|  | (0.0146) | (0.00200) | (0.00148) | (0.00148) | (0.00124) | (0.00124) |
| Group*Treat | 0.317*** | 0.317* | 0.435*** | 0.438*** | 1.016*** | 0.986*** |
|  | (0.0559) | (0.164) | (0.107) | (0.107) | (0.102) | (0.0996) |
| ATE | 0.687*** | 0.687*** | 0.720*** | 0.721*** | 0.687*** | 0.687*** |
|  | (0.0685) | (0.121) | (0.0750) | (0.0750) | (0.0731) | (0.0731) |
| Constant | 0.0306*** | 0.0306*** | -0.0507*** | 0.0119 | 0.416*** | 0.229*** |
|  | (0.00860) | (0.00154) | (0.00848) | (0.0992) | (0.00436) | (0.00500) |
| Observations | 1,349,280 | 1,349,280 | 1,349,280 | 1,349,280 | 1,349,280 | 1,349,280 |
| R-squared | 0.274 | 0.274 | 0.345 | 0.346 | 0.032 | . |
| Firm clustered s.e | No | No | Yes | Yes | Yes | Yes |
| Industry clustered s.e | No | Yes | No | No | No | No |
| Firm FE | No | No | No | No | Yes | No |
| Firm RE | No | No | No | No | No | Yes |
| Industry FE | No | No | Yes | Yes | No | No |
| Municip FE | No | No | No | Yes | No | No |

Notes. Dependent variable: Number of 19-25-year-olds. Treatment period: 2006-2008. Underlying time period: 2003-2005. Only surviving firms with at least one employee per year are included. The point estimates in the figures are represented by the ATE estimate in the fifth column. DDD estimation.

*p<0.1, **p<0.05, ***p<0.01

**Table A4.** 2006-2008 employment effect for older individuals by treatment intensity. DDD regressions.

| **Specification:** | **1** | **2** | **3** | **4** | **5** | **6** | **Specification:** | **1** | **2** | **3** | **4** | **5** | **6** |
| --- | --- | --- | --- | --- | --- | --- | --- | --- | --- | --- | --- | --- | --- |
| **Tr. intensity:** | *0-20* | *0-20* | *0-20* | *0-20* | *0-20* | *0-20* | **Tr. intensity:** | *20-40* | *20-40* | *20-40* | *20-40* | *20-40* | *20-40* |
| Time | 0.0857*** | 0.0857*** | 0.0829*** | 0.0853*** | 0.0857*** | 0.0857*** | Time | 0.0857*** | 0.0857*** | 0.0832*** | 0.0857*** | 0.0857*** | 0.0857*** |
|  | (0.0185) | (0.0108) | (0.00365) | (0.00367) | (0.00343) | (0.00343) |  | (0.0179) | (0.0108) | (0.00367) | (0.00368) | (0.00343) | (0.00343) |
| Group | 4.251*** | 4.251*** | 3.815*** | 3.794*** | -0.0984 | 0.138** | Group | 4.552*** | 4.552*** | 4.099*** | 4.074*** | -0.251*** | 0.0429 |
|  | (0.0714) | (0.528) | (0.174) | (0.173) | (0.0709) | (0.0689) |  | (0.0691) | (0.450) | (0.141) | (0.141) | (0.0626) | (0.0608) |
| Treat | -0.0850*** | -0.0850*** | -0.0627*** | -0.0626*** | 0.0461*** | 0.0428*** | Treat | -0.0850*** | -0.0850*** | -0.0659*** | -0.0654*** | 0.0468*** | 0.0434*** |
|  | (0.0210) | (0.0236) | (0.0135) | (0.0135) | (0.00542) | (0.00537) |  | (0.0204) | (0.0236) | (0.0135) | (0.0135) | (0.00542) | (0.00537) |
| Group*Time | 0.267*** | 0.267*** | 0.255*** | 0.254*** | 0.267*** | 0.267*** | Group*Time | 0.252*** | 0.252*** | 0.231*** | 0.227*** | 0.252*** | 0.252*** |
|  | (0.0874) | (0.0502) | (0.0348) | (0.0348) | (0.0345) | (0.0345) |  | (0.0846) | (0.0627) | (0.0349) | (0.0350) | (0.0338) | (0.0338) |
| Time*Treat | 0.0193 | 0.0193 | 0.0179*** | 0.0169*** | 0.0193*** | 0.0193*** | Time*Treat | 0.0193 | 0.0193 | 0.0183*** | 0.0171*** | 0.0193*** | 0.0193*** |
|  | (0.0257) | (0.0124) | (0.00509) | (0.00510) | (0.00478) | (0.00478) |  | (0.0249) | (0.0124) | (0.00509) | (0.00510) | (0.00478) | (0.00478) |
| Group*Treat | 0.361*** | 0.361 | 0.292 | 0.275 | 0.266*** | 0.252*** | Group*Treat | 0.355*** | 0.355 | 0.332* | 0.320* | 0.519*** | 0.484*** |
|  | (0.0982) | (0.276) | (0.232) | (0.232) | (0.0835) | (0.0819) |  | (0.0952) | (0.245) | (0.193) | (0.193) | (0.0775) | (0.0758) |
| ATE | 0.0351 | 0.0351 | 0.0403 | 0.0372 | 0.0351 | 0.0351 | ATE | 0.0453 | 0.0453 | 0.0687 | 0.0722 | 0.0453 | 0.0453 |
|  | (0.120) | (0.0962) | (0.0497) | (0.0497) | (0.0486) | (0.0486) |  | (0.117) | (0.0868) | (0.0491) | (0.0491) | (0.0480) | (0.0480) |
| Constant | 2.595*** | 2.595*** | 1.129*** | 1.721*** | 2.728*** | 2.792*** | Constant | 2.595*** | 2.595*** | 1.112*** | 1.651*** | 2.743*** | 2.811*** |
|  | (0.0151) | (0.107) | (0.0163) | (0.197) | (0.00443) | (0.0136) |  | (0.0146) | (0.107) | (0.0174) | (0.187) | (0.00433) | (0.0131) |
| Observations | 1,349,253 | 1,349,253 | 1,349,253 | 1,349,253 | 1,349,253 | 1,349,253 | Observations | 1,349,322 | 1,349,322 | 1,349,322 | 1,349,322 | 1,349,322 | 1,349,322 |
| R-squared | 0.020 | 0.020 | 0.127 | 0.132 | 0.003 | . | R-squared | 0.023 | 0.023 | 0.150 | 0.155 | 0.003 | . |
| Firm clustered s.e | No | No | Yes | Yes | Yes | Yes | Firm clustered s.e | No | No | Yes | Yes | Yes | Yes |
| Industry clustered s.e | No | Yes | No | No | No | No | Industry clustered s.e | No | Yes | No | No | No | No |
| Firm FE | No | No | No | No | Yes | No | Firm FE | No | No | No | No | Yes | No |
| Firm RE | No | No | No | No | No | Yes | Firm RE | No | No | No | No | No | Yes |
| Industry FE | No | No | Yes | Yes | No | No | Industry FE | No | No | Yes | Yes | No | No |
| Municip FE | No | No | No | Yes | No | No | Municip FE | No | No | No | Yes | No | No |

Notes. Dependent variable: Number of employees over the age of 25. Treatment period: 2006-2008. Underlying time period: 2003-2005. Only surviving firms with at least one employee per year are included. The point estimates in the figures are represented by the ATE estimate in the fifth column. DDD estimation. *p<0.1, **p<0.05, ***p<0.01

**Cont. - Table A4.** 2006-2008 employment effect for older individuals by treatment intensity. DDD regressions.

| **Specification:** | **1** | **2** | **3** | **4** | **5** | **6** | **Specification:** | **1** | **2** | **3** | **4** | **5** | **6** |
| --- | --- | --- | --- | --- | --- | --- | --- | --- | --- | --- | --- | --- | --- |
| **Tr. intensity:** | *40-60* | *40-60* | *40-60* | *40-60* | *40-60* | *40-60* | **Tr. intensity:** | *60-80* | *60-80* | *60-80* | *60-80* | *60-80* | *60-80* |
| Time | 0.0857*** | 0.0857*** | 0.0830*** | 0.0856*** | 0.0857*** | 0.0857*** | Time | 0.0857*** | 0.0857*** | 0.0814*** | 0.0841*** | 0.0857*** | 0.0857*** |
|  | (0.0190) | (0.0108) | (0.00363) | (0.00364) | (0.00343) | (0.00343) |  | (0.0224) | (0.0108) | (0.00377) | (0.00379) | (0.00343) | (0.00343) |
| Group | 6.566*** | 6.566*** | 5.830*** | 5.803*** | -0.333*** | 0.0501 | Group | 12.17*** | 12.17*** | 11.27*** | 11.21*** | -0.0732 | 0.764*** |
|  | (0.0732) | (0.499) | (0.165) | (0.165) | (0.0747) | (0.0727) |  | (0.0865) | (0.904) | (0.276) | (0.276) | (0.129) | (0.125) |
| Treat | -0.0850*** | -0.0850*** | -0.0668*** | -0.0654*** | 0.0473*** | 0.0441*** | Treat | -0.0850*** | -0.0850*** | -0.0731*** | -0.0727*** | 0.0480*** | 0.0452*** |
|  | (0.0215) | (0.0236) | (0.0135) | (0.0135) | (0.00545) | (0.00541) |  | (0.0255) | (0.0236) | (0.0137) | (0.0137) | (0.00549) | (0.00545) |
| Group*Time | 0.265*** | 0.265*** | 0.257*** | 0.255*** | 0.265*** | 0.265*** | Group*Time | 0.409*** | 0.409*** | 0.382*** | 0.380*** | 0.409*** | 0.409*** |
|  | (0.0896) | (0.0606) | (0.0361) | (0.0361) | (0.0358) | (0.0358) |  | (0.106) | (0.124) | (0.0539) | (0.0537) | (0.0531) | (0.0531) |
| Time*Treat | 0.0193 | 0.0193 | 0.0195*** | 0.0183*** | 0.0193*** | 0.0193*** | Time*Treat | 0.0193 | 0.0193 | 0.0205*** | 0.0193*** | 0.0193*** | 0.0193*** |
|  | (0.0264) | (0.0124) | (0.00508) | (0.00510) | (0.00478) | (0.00478) |  | (0.0312) | (0.0124) | (0.00527) | (0.00528) | (0.00478) | (0.00478) |
| Group*Treat | 0.0522 | 0.0522 | 0.199 | 0.202 | 0.786*** | 0.767*** | Group*Treat | -0.936*** | -0.936** | -0.815** | -0.809** | 1.149*** | 1.105*** |
|  | (0.101) | (0.292) | (0.233) | (0.233) | (0.0890) | (0.0876) |  | (0.119) | (0.406) | (0.347) | (0.347) | (0.124) | (0.123) |
| ATE | 0.0532 | 0.0532 | 0.0572 | 0.0599 | 0.0532 | 0.0532 | ATE | 0.157 | 0.157 | 0.183** | 0.185*** | 0.157** | 0.157** |
|  | (0.123) | (0.0867) | (0.0505) | (0.0506) | (0.0499) | (0.0499) |  | (0.146) | (0.160) | (0.0716) | (0.0714) | (0.0696) | (0.0696) |
| Constant | 2.595*** | 2.595*** | 1.077*** | 1.704*** | 2.824*** | 2.894*** | Constant | 2.595*** | 2.595*** | 1.086*** | 1.591*** | 3.036*** | 3.119*** |
|  | (0.0155) | (0.107) | (0.0186) | (0.233) | (0.00462) | (0.0139) |  | (0.0183) | (0.107) | (0.0202) | (0.196) | (0.00589) | (0.0168) |
| Observations | 1,349,250 | 1,349,250 | 1,349,250 | 1,349,250 | 1,349,250 | 1,349,250 | Observations | 1,349,340 | 1,349,340 | 1,349,340 | 1,349,340 | 1,349,340 | 1,349,340 |
| R-squared | 0.039 | 0.039 | 0.157 | 0.161 | 0.004 | . | R-squared | 0.083 | 0.083 | 0.188 | 0.192 | 0.008 | . |
| Firm clustered s.e | No | No | Yes | Yes | Yes | Yes | Firm clustered s.e | No | No | Yes | Yes | Yes | Yes |
| Industry clustered s.e | No | Yes | No | No | No | No | Industry clustered s.e | No | Yes | No | No | No | No |
| Firm FE | No | No | No | No | Yes | No | Firm FE | No | No | No | No | Yes | No |
| Firm RE | No | No | No | No | No | Yes | Firm RE | No | No | No | No | No | Yes |
| Industry FE | No | No | Yes | Yes | No | No | Industry FE | No | No | Yes | Yes | No | No |
| Municip FE | No | No | No | Yes | No | No | Municip FE | No | No | No | Yes | No | No |

Notes. Dependent variable: Number of employees over the age of 25. Treatment period: 2006-2008. Underlying time period: 2003-2005. Only surviving firms with at least one employee per year are included. The point estimates in the figures are represented by the ATE estimate in the fifth column. DDD estimation. *p<0.1, **p<0.05, ***p<0.01

**Cont. - Table A4.** 2006-2008 employment effect for older individuals by treatment intensity. DDD regressions.

| **Specification:** | **1** | **2** | **3** | **4** | **5** | **6** |
| --- | --- | --- | --- | --- | --- | --- |
| **Tr. intensity:** | *80-100* | *80-100* | *80-100* | *80-100* | *80-100* | *80-100* |
| Time | 0.0857 | 0.0857*** | 0.0734*** | 0.0746*** | 0.0857*** | 0.0857*** |
|  | (0.0906) | (0.0108) | (0.00707) | (0.00714) | (0.00343) | (0.00343) |
| Group | 64.31*** | 64.31*** | 52.71*** | 52.61*** | 2.731*** | 5.528*** |
|  | (0.350) | (10.58) | (1.089) | (1.090) | (0.471) | (0.459) |
| Treat | -0.0850 | -0.0850*** | -0.167*** | -0.156*** | 0.0410*** | 0.0388*** |
|  | (0.103) | (0.0236) | (0.0275) | (0.0277) | (0.00563) | (0.00562) |
| Group*Time | 1.017** | 1.017 | 0.818*** | 0.819*** | 1.017*** | 1.017*** |
|  | (0.428) | (0.879) | (0.195) | (0.195) | (0.115) | (0.115) |
| Time*Treat | 0.0193 | 0.0193 | 0.0243** | 0.0238** | 0.0193*** | 0.0193*** |
|  | (0.126) | (0.0124) | (0.0102) | (0.0102) | (0.00478) | (0.00478) |
| Group*Treat | -5.970*** | -5.970*** | -3.730*** | -3.707*** | 3.157*** | 3.119*** |
|  | (0.482) | (1.710) | (0.653) | (0.652) | (0.261) | (0.260) |
| ATE | 0.206 | 0.206 | 0.587** | 0.597** | 0.206 | 0.206 |
|  | (0.590) | (1.326) | (0.274) | (0.274) | (0.149) | (0.149) |
| Constant | 2.595*** | 2.595*** | 1.037*** | 1.010* | 5.127*** | 4.610*** |
|  | (0.0740) | (0.107) | (0.0534) | (0.582) | (0.0193) | (0.0556) |
| Observations | 1,349,280 | 1,349,280 | 1,349,280 | 1,349,280 | 1,349,280 | 1,349,280 |
| R-squared | 0.129 | 0.129 | 0.338 | 0.339 | 0.021 | . |
| Firm clustered s.e | No | No | Yes | Yes | Yes | Yes |
| Industry clustered s.e | No | Yes | No | No | No | No |
| Firm FE | No | No | No | No | Yes | No |
| Firm RE | No | No | No | No | No | Yes |
| Industry FE | No | No | Yes | Yes | No | No |
| Municip FE | No | No | No | Yes | No | No |

Notes. Dependent variable: Number of employees over the age of 25. Treatment period: 2006-2008. Underlying time period: 2003-2005. Only surviving firms with at least one employee per year are included. The point estimates in the figures are represented by the ATE estimate in the fifth column. DDD estimation. *p<0.1, **p<0.05, ***p<0.01

**Table A5.** 2006-2008 wage effect for incumbent employees with a minimum age of 26 by treatment intensity. DDD regressions.

| **Specification:** | **1** | **2** | **3** | **4** | **5** | **6** | **Specification:** | **1** | **2** | **3** | **4** | **5** | **6** |
| --- | --- | --- | --- | --- | --- | --- | --- | --- | --- | --- | --- | --- | --- |
| **Tr. intensity:** | *0-20* | *0-20* | *0-20* | *0-20* | *0-20* | *0-20* | **Tr. intensity:** | *20-40* | *20-40* | *20-40* | *20-40* | *20-40* | *20-40* |
| Time | 420.7*** | 420.7*** | 413.1*** | 420.5*** | 420.7*** | 420.7*** | Time | 420.7*** | 420.7*** | 413.5*** | 420.8*** | 420.7*** | 420.7*** |
|  | (50.19) | (28.46) | (5.216) | (5.318) | (3.995) | (3.995) |  | (49.72) | (28.46) | (4.945) | (5.052) | (3.995) | (3.995) |
| Group | 10,577*** | 10,577*** | 9,708*** | 9,670*** | -124.7 | 254.1 | Group | 10,565*** | 10,565*** | 9,781*** | 9,706*** | -576.4*** | -137.3 |
|  | (208.3) | (1,187) | (397.5) | (392.7) | (212.0) | (204.7) |  | (201.9) | (1,097) | (389.9) | (389.0) | (179.6) | (174.3) |
| Treat | 301.5*** | 301.5*** | 292.4*** | 285.1*** | 566.4*** | 561.9*** | Treat | 301.5*** | 301.5*** | 287.9*** | 281.8*** | 569.0*** | 564.8*** |
|  | (57.16) | (70.10) | (37.24) | (37.19) | (15.68) | (15.57) |  | (56.63) | (70.10) | (37.12) | (37.05) | (15.70) | (15.59) |
| Group*Time | 602.9** | 602.9*** | 576.3*** | 574.1*** | 602.9*** | 602.9*** | Group*Time | 612.7** | 612.7*** | 557.1*** | 548.1*** | 612.7*** | 612.7*** |
|  | (255.1) | (111.7) | (41.12) | (41.09) | (38.16) | (38.16) |  | (247.3) | (87.89) | (42.19) | (42.59) | (34.06) | (34.06) |
| Time*Treat | -186.3*** | -186.3*** | -190.2*** | -193.3*** | -186.3*** | -186.3*** | Time*Treat | -186.3*** | -186.3*** | -187.8*** | -191.2*** | -186.3*** | -186.3*** |
|  | (70.01) | (27.94) | (7.248) | (7.332) | (5.444) | (5.444) |  | (69.36) | (27.94) | (6.987) | (7.067) | (5.444) | (5.444) |
| Group*Treat | 1,137*** | 1,137* | 963.4* | 906.4 | 475.9** | 441.5* | Group*Treat | 915.7*** | 915.7 | 1,016* | 988.1* | 1,404*** | 1,320*** |
|  | (286.6) | (681.9) | (555.9) | (553.6) | (240.3) | (234.0) |  | (278.6) | (698.0) | (531.9) | (530.1) | (232.4) | (227.6) |
| ATE | 99.11 | 99.11 | 110.8* | 108.4* | 99.11* | 99.11* | ATE | 136.8 | 136.8 | 204.5*** | 211.7*** | 136.8*** | 136.8*** |
|  | (351.0) | (237.9) | (59.38) | (59.24) | (52.96) | (52.96) |  | (341.2) | (128.4) | (58.48) | (58.86) | (50.55) | (50.55) |
| Constant | 5,710*** | 5,710*** | 1,724*** | 4,154*** | 6,015*** | 6,057*** | Constant | 5,710*** | 5,710*** | 1,733*** | 3,820*** | 6,023*** | 6,077*** |
|  | (40.98) | (330.6) | (44.76) | (734.1) | (10.57) | (35.81) |  | (40.60) | (330.6) | (46.76) | (676.6) | (10.86) | (35.23) |
| Observations | 1,247,634 | 1,247,634 | 1,247,634 | 1,247,634 | 1,247,634 | 1,247,634 | Observations | 1,249,608 | 1,249,608 | 1,249,608 | 1,249,608 | 1,249,608 | 1,249,608 |
| R-squared | 0.016 | 0.016 | 0.138 | 0.145 | 0.010 | . | R-squared | 0.016 | 0.016 | 0.140 | 0.147 | 0.012 | . |
| Firm clustered s.e | No | No | Yes | Yes | Yes | Yes | Firm clustered s.e | No | No | Yes | Yes | Yes | Yes |
| Industry clustered s.e | No | Yes | No | No | No | No | Industry clustered s.e | No | Yes | No | No | No | No |
| Firm FE | No | No | No | No | Yes | No | Firm FE | No | No | No | No | Yes | No |
| Firm RE | No | No | No | No | No | Yes | Firm RE | No | No | No | No | No | Yes |
| Industry FE | No | No | Yes | Yes | No | No | Industry FE | No | No | Yes | Yes | No | No |
| Municip FE | No | No | No | Yes | No | No | Municip FE | No | No | No | Yes | No | No |

Notes. Dependent variable: Gross wage sum for incumbent employees of minimum age 26 (measured in 100 SEK). Treatment period: 2006-2008. Underlying time period: 2003-2005. Only surviving firms with at least one employee per year are included. The point estimates in the figures are represented by the ATE estimate in the fifth column. DDD estimation.

*p<0.1, **p<0.05, ***p<0.01

**Cont. - Table A5.** 2006-2008 wage effect for incumbent employees with a minimum age of 26 by treatment intensity. DDD regressions.

| **Specification:** | **1** | **2** | **3** | **4** | **5** | **6** | **Specification:** | **1** | **2** | **3** | **4** | **5** | **6** |
| --- | --- | --- | --- | --- | --- | --- | --- | --- | --- | --- | --- | --- | --- |
| **Tr. intensity:** | *40-60* | *40-60* | *40-60* | *40-60* | *40-60* | *40-60* | **Tr. intensity:** | *60-80* | *60-80* | *60-80* | *60-80* | *60-80* | *60-80* |
| Time | 420.7*** | 420.7*** | 413.7*** | 421.2*** | 420.7*** | 420.7*** | Time | 420.7*** | 420.7*** | 409.8*** | 417.5*** | 420.7*** | 420.7*** |
|  | (52.89) | (28.46) | (4.989) | (5.103) | (3.995) | (3.995) |  | (58.74) | (28.46) | (5.273) | (5.388) | (3.995) | (3.995) |
| Group | 15,032*** | 15,032*** | 13,378*** | 13,298*** | -812.8*** | -265.3 | Group | 27,324*** | 27,324*** | 25,446*** | 25,250*** | -942.4** | 426.7 |
|  | (207.3) | (1,180) | (418.6) | (417.5) | (213.9) | (208.3) |  | (227.0) | (2,122) | (650.1) | (648.3) | (366.4) | (353.4) |
| Treat | 301.5*** | 301.5*** | 288.0*** | 283.4*** | 573.1*** | 569.0*** | Treat | 301.5*** | 301.5*** | 277.5*** | 270.0*** | 577.4*** | 573.4*** |
|  | (60.23) | (70.10) | (37.30) | (37.24) | (15.80) | (15.70) |  | (66.90) | (70.10) | (37.41) | (37.32) | (16.01) | (15.91) |
| Group*Time | 840.6*** | 840.6*** | 826.9*** | 818.1*** | 840.6*** | 840.6*** | Group*Time | 1,581*** | 1,581*** | 1,520*** | 1,512*** | 1,581*** | 1,581*** |
|  | (253.9) | (168.9) | (38.66) | (38.82) | (35.92) | (35.92) |  | (278.0) | (264.0) | (55.33) | (55.92) | (50.60) | (50.60) |
| Time*Treat | -186.3** | -186.3*** | -187.3*** | -190.4*** | -186.3*** | -186.3*** | Time*Treat | -186.3** | -186.3*** | -185.2*** | -188.4*** | -186.3*** | -186.3*** |
|  | (73.77) | (27.94) | (7.064) | (7.157) | (5.444) | (5.444) |  | (81.94) | (27.94) | (7.449) | (7.551) | (5.444) | (5.444) |
| Group*Treat | 415.6 | 415.6 | 898.9 | 920.3 | 2,014*** | 1,980*** | Group*Treat | -2,050*** | -2,050** | -1,616** | -1,594** | 3,621*** | 3,518*** |
|  | (286.4) | (737.4) | (615.0) | (614.2) | (260.1) | (255.7) |  | (313.1) | (943.6) | (797.7) | (796.1) | (357.1) | (351.2) |
| ATE | 36.13 | 36.13 | 50.08 | 59.63 | 36.13 | 36.13 | ATE | -212.2 | -212.2 | -168.8** | -162.8** | -212.2*** | -212.2*** |
|  | (350.8) | (215.1) | (55.09) | (55.33) | (51.05) | (51.05) |  | (383.5) | (291.0) | (73.44) | (73.81) | (64.61) | (64.61) |
| Constant | 5,710*** | 5,710*** | 1,628*** | 4,002*** | 6,235*** | 6,295*** | Constant | 5,710*** | 5,710*** | 1,675*** | 3,807*** | 6,725*** | 6,803*** |
|  | (43.18) | (330.6) | (48.70) | (769.2) | (11.86) | (37.97) |  | (47.96) | (330.6) | (49.63) | (691.2) | (16.78) | (43.49) |
| Observations | 1,253,385 | 1,253,385 | 1,253,385 | 1,253,385 | 1,253,385 | 1,253,385 | Observations | 1,255,272 | 1,255,272 | 1,255,272 | 1,255,272 | 1,255,272 | 1,255,272 |
| R-squared | 0.029 | 0.029 | 0.150 | 0.156 | 0.013 | . | R-squared | 0.068 | 0.068 | 0.176 | 0.182 | 0.019 | . |
| Firm clustered s.e | No | No | Yes | Yes | Yes | Yes | Firm clustered s.e | No | No | Yes | Yes | Yes | Yes |
| Industry clustered s.e | No | Yes | No | No | No | No | Industry clustered s.e | No | Yes | No | No | No | No |
| Firm FE | No | No | No | No | Yes | No | Firm FE | No | No | No | No | Yes | No |
| Firm RE | No | No | No | No | No | Yes | Firm RE | No | No | No | No | No | Yes |
| Industry FE | No | No | Yes | Yes | No | No | Industry FE | No | No | Yes | Yes | No | No |
| Municip FE | No | No | No | Yes | No | No | Municip FE | No | No | No | Yes | No | No |

Notes. Dependent variable: Gross wage sum for incumbent employees of minimum age 26 (measured in 100 SEK). Treatment period: 2006-2008. Underlying time period: 2003-2005. Only surviving firms with at least one employee per year are included. The point estimates in the figures are represented by the ATE estimate in the fifth column. DDD estimation.

*p<0.1, **p<0.05, ***p<0.01

**Cont. - Table A5.** 2006-2008 wage effect for incumbent employees with a minimum age of 26 by treatment intensity. DDD regressions.

| **Specification:** | **1** | **2** | **3** | **4** | **5** | **6** |
| --- | --- | --- | --- | --- | --- | --- |
| **Tr. intensity:** | *80-100* | *80-100* | *80-100* | *80-100* | *80-100* | *80-100* |
| Time | 420.7*** | 420.7*** | 402.6*** | 408.6*** | 420.7*** | 420.7*** |
|  | (127.7) | (28.46) | (8.314) | (8.567) | (3.995) | (3.995) |
| Group | 102,773*** | 102,773*** | 91,957*** | 91,597*** | 3,178*** | 10,809*** |
|  | (489.5) | (10,601) | (1,684) | (1,682) | (1,220) | (1,134) |
| Treat | 301.5** | 301.5*** | 203.4*** | 210.2*** | 550.5*** | 544.6*** |
|  | (145.4) | (70.10) | (45.60) | (45.61) | (16.52) | (16.44) |
| Group*Time | 5,040*** | 5,040*** | 4,788*** | 4,795*** | 5,040*** | 5,040*** |
|  | (599.6) | (1,578) | (190.9) | (190.4) | (105.8) | (105.8) |
| Time*Treat | -186.3 | -186.3*** | -185.0*** | -188.9*** | -186.3*** | -186.3*** |
|  | (178.1) | (27.94) | (13.43) | (13.57) | (5.444) | (5.444) |
| Group*Treat | -7,380*** | -7,380*** | -5,250*** | -5,167*** | 9,679*** | 9,559*** |
|  | (674.0) | (2,775) | (1,176) | (1,174) | (565.3) | (563.2) |
| ATE | -665.3 | -665.3 | -291.5 | -294.6 | -665.3*** | -665.3*** |
|  | (825.5) | (1,618) | (272.9) | (271.5) | (103.2) | (103.2) |
| Constant | 5,710*** | 5,710*** | 1,599*** | 4,267*** | 9,808*** | 8,863*** |
|  | (104.3) | (330.6) | (88.23) | (1,538) | (53.47) | (89.14) |
| Observations | 1,256,490 | 1,256,490 | 1,256,490 | 1,256,490 | 1,256,490 | 1,256,490 |
| R-squared | 0.180 | 0.180 | 0.305 | 0.307 | 0.053 | . |
| Firm clustered s.e | No | No | Yes | Yes | Yes | Yes |
| Industry clustered s.e | No | Yes | No | No | No | No |
| Firm FE | No | No | No | No | Yes | No |
| Firm RE | No | No | No | No | No | Yes |
| Industry FE | No | No | Yes | Yes | No | No |
| Municip FE | No | No | No | Yes | No | No |

Notes. Dependent variable: Gross wage sum for incumbent employees of minimum age 26 (measured in 100 SEK). Treatment period: 2006-2008. Underlying time period: 2003-2005. Only surviving firms with at least one employee per year are included. The point estimates in the figures are represented by the ATE estimate in the fifth column. DDD estimation.

*p<0.1, **p<0.05, ***p<0.01
